# A multi-omics analysis of the grapevine pathogen *Lasiodiplodia theobromae* reveals that temperature affects the expression of virulence- and pathogenicity-related genes

**DOI:** 10.1038/s41598-019-49551-w

**Published:** 2019-09-11

**Authors:** Carina Félix, Rodrigo Meneses, Micael F. M. Gonçalves, Laurentijn Tilleman, Ana S. Duarte, Jesus V. Jorrín-Novo, Yves Van de Peer, Dieter Deforce, Filip Van Nieuwerburgh, Ana C. Esteves, Artur Alves

**Affiliations:** 1 Department of Biology, CESAM, University of Aveiro, Campus Universitário de Santiago, 3810-193 Aveiro, Portugal; 2 Department of Plant Biotechnology and Bioinformatics, Ghent University, 9052 Ghent, Belgium; 3 Laboratory of Pharmaceutical Biotechnology, Ghent University, Ottergemsesteenweg 460, B-9000 Ghent, Belgium; 4 Agroforestry and Plant Biochemistry, Proteomics and Systems Biology, Department of Biochemistry and Molecular Biology, Universidad de Córdoba, Córdoba, Spain; 5 Center for Plant Systems Biology, VIB, 9052 Ghent, Belgium; 6 Department of Biochemistry, Genetics and Microbiology, University of Pretoria, Pretoria, 0028 South Africa; 7 Present Address: Portuguese Catholic University, Health Science Institute-Viseu, Estrada da Circunvalação, 3504-505 Viseu, Portugal

**Keywords:** Fungi, Pathogens

## Abstract

*Lasiodiplodia theobromae* (Botryosphaeriaceae, Ascomycota) is a plant pathogen and human opportunist whose pathogenicity is modulated by temperature. The molecular effects of temperature on *L*. *theobromae* are mostly unknown, so we used a multi-omics approach to understand how temperature affects the molecular mechanisms of pathogenicity. The genome of *L*. *theobromae* LA-SOL3 was sequenced (Illumina MiSeq) and annotated. Furthermore, the transcriptome (Illumina TruSeq) and proteome (Orbitrap LC-MS/MS) of LA-SOL3 grown at 25 °C and 37 °C were analysed. Proteins related to pathogenicity (plant cell wall degradation, toxin synthesis, mitogen-activated kinases pathway and proteins involved in the velvet complex) were more abundant when the fungus grew at 25 °C. At 37 °C, proteins related to pathogenicity were less abundant than at 25 °C, while proteins related to cell wall organisation were more abundant. On the other hand, virulence factors involved in human pathogenesis, such as the SSD1 virulence protein, were expressed only at 37 °C. Taken together, our results showed that this species presents a typical phytopathogenic molecular profile that is compatible with a hemibiotrophic lifestyle. We showed that *L*. *theobromae* is equipped with the pathogenesis toolbox that enables it to infect not only plants but also animals.

## Introduction

*Lasiodiplodia theobromae* (Pat.) Griffon & Maubl. (Botryosphaeriaceae, Ascomycota), an emergent grapevine trunk disease agent, is frequently the most commonly found species in vineyards, as well as the most aggressive, especially in warmer climates^1,2^.

This pathogen has been associated with almost 500 plant hosts and a large number of diseases, especially in crops^3^. In humans, *L*. *theobromae* has also been associated with several human infections, such as ocular infections, but sometimes leading to death^4–8^.

Changes in the climate, namely increasing temperature, can influence the behaviour of pathogens^9^. Nonetheless, the impact of these alterations on microbial pathogen/host interactions, that ultimately can lead changes in virulence, has barely been addressed^10–12^.

It has been suggested that the expression of virulence factors in *L*. *theobromae* can be modulated by temperature^13,14^. Úrbez-Torres *et al*.^1^ and later Yan *et al*.^14^ showed that the larger lesions caused by *L*. *theobromae* on grapevines were observed when the plants were grown at 35 °C. However, it is unclear if this results from increased virulence of the pathogen or increased susceptibility of the host due to heat stress effect.

An approach integrating multiple omics technologies can be used to understand the functional principles and the dynamics of cellular systems^15,16^. In the last years, some studies in species of the family Botryosphaeriaceae, including *L*. *theobromae*, revealed important findings during *in vitro* short-term heat stress response. It has been argued that high temperatures induced the expansion of gene families associated with cell wall degradation, nutrient uptake, secondary metabolism, membrane transport and also genes related to carbohydrate-binding modules, glycosyl hydrolase families and lysine motif domain^14^. Also, the temperature was found to up-regulate genes encoding for salicylic acid and plant phenylpropanoid precursors-degrading enzymes^17^.

Sequencing and annotation of a pathogen’s genome open the possibility of performing multi-omics approaches and, identify gene and gene products that are affected by temperature. The first genome of *L*. *theobromae* was also recently released^14^. However, none of the studies used a multi-omics integrated approach for a more detailed, comprehensive characterisation of the species.

We aimed to understand how the expression of pathogenicity and virulence-related genes of *L*. *theobromae* is affected by its *in vitro* growth temperature. For this, we integrated genome sequencing and RNA-seq with proteomics data to study the molecular basis of *L*. *theobromae* pathogenicity at different temperatures.

## Results

### Genome sequencing, assembly and annotation

*Lasiodioplodia theobromae* LA-SOL3 genome was sequenced into 38.8 million matched paired-end reads by Illumina sequencing (Table 1). Assembly led to a genome size of 43.9 Mb (43,925,482 bp) with approximately 214 x coverage, divided into 413 scaffolds with a minimum size of 2 Mb, a total of 79 gaps and 2,368 Ns (undetermined nucleotides), as well as an overall G + C content of 54.75%, similar to the described earlier for *L*. *theobromae* CSS-01s^14^. The N50 value was approximately 249 kb. RepeatMasker analysis, coupled with RepeatModeler and RepeatProteinMask, discovered an overall repeat content of 2.21% of the genome. Some characteristics of *L*. *theobromae* LA-SOL3 genome are shown in Table 1.

**Table 1:** General statistics of *L*. *theobromae* LA-SOL3 genome assembly, gene prediction and comparison with the available genome of *L*. *theobromae* CSS-01s (MDYX00000000.1).

*Lasiodiplodia theobromae*	LA-SOL3	CSS-01s (MDYX00000000.1)
Size (Mb)	43.9	43.3
Coverage	214x	90x
% G + C content	54.75	54.77
% Repeats	2.21	3.02
Number of genes	12785	12902
Average gene length (bp)	1610	1629
% of genome covered by genes	46.8	~48.5
% of genome covered by CDS	42.7	43.6
Gene density (genes/Mb)	291	297
Average exons per mRNA	2.8	2.8
Average exon length (bp)	510	506
Average introns per mRNA	1.8	1.8
Average intron length (bp)	86	88
Average mRNAs per gene	1	n.a.

BRAKER software predicted 12,785 genes, with an average length of 1,610 bp, a gene density of 291 genes/Mb and a total of 46.8% of the genome covered by protein-coding genes. There is an average relation of 1 mRNA per gene, with an average of 2.8 exons and 1.8 introns per mRNA, per gene.

*Lasiodiplodia theobromae* LA-SOL3 genome encodes 667 extracellular proteins. From these, 7.3% (335) of all enzymes encoded by the genome are enzymes. While the most represented enzymes have oxidoreductase (1299 in total, 46 secreted), transferase (962 in total, 7 secreted) or hydrolase (1486 in total) functions, secreted enzymes are mainly hydrolases (237). Other enzymes were also identified, such as lyases (291 in total, 28 secreted), isomerases (119 in total, 10 secreted) and ligases (192 in total), although some of the predicted genes were classified in more than one enzyme category.

**Figure 1:**
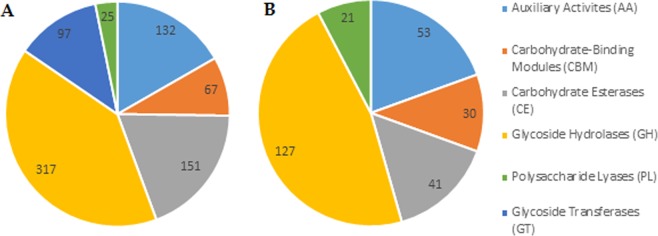
Predicted CAZymes (**A**) and predicted secreted CAZymes (**B**) in the genome of *L*. *theobromae* LA-SOL3.

Quite a number of genes related to pathogenicity and virulence were identified. Among them are oxidoreductases and CAZymes. Oxidoreductases are involved in plant pathogenesis accounting for the degradation of lignin and the detoxification of host defence reactive oxygen species^18^, while CAZymes participate in the colonisation and infection of plant pathogenic fungi, disassembling the plant cell wall^14,19^. In total, *L*. *theobromae* LA-SOL3 genome encodes for 1299 oxidoreductases (46 are predicted to be extracellular) and 789 CAZymes (272 are predicted to be extracellular). Glycoside hydrolases are the CAZymes with most representatives, accounting for 317 proteins (127 secreted, Fig. 1).

Among oxidoreductases, forty genes encode peroxidases, with a total of 69 functions (Table S1). The number of functions was higher than the number of proteins since 14 genes were annotated with two or more peroxidase types.

Secondary gene clusters are responsible for secondary metabolites synthesis. These metabolites are, along with proteins, involved in the development of disease symptoms and, sometimes, in host death. *Lasiodiplodia theobromae* LA-SOL3 genome encodes 52 secondary metabolite gene clusters – per type: 8 terpenes, 17 t1pks (type 1 polyketide synthases), 11 nrps (nonribosomal peptide-synthetase), 6 t1pks-nrps and 10 “other”. Of these, 7 clusters were found to have known homology to other described clusters: NRPS – chaetocin (13%) and hexadehydro-astechrome (HAS) (25%); terpene – PR toxin (50%); T1PKS – brefeldin (20%), emericellin (50%) and lasiodiplodin (71%) and T1PKS-NRPS – fusaridone A (12%).

In pathogenic fungi, cytochrome P450 enzymes are usually related to their defence against toxic substances produced by the hosts. In fact, it is known that P450 is involved in the production of mycotoxins (*e*.*g*. aflatoxins), and gibberellins^20^. A total of 43 genes were annotated as coding for fungal cytochrome P450, within 23 different P450 families (Table S2).

Several studies suggest that transporters play a crucial role in fungal pathogenesis, contributing to different functions such as drugs secretion or the transport of molecules involved in appressoria formation^14^. In the genome of *L*. *theobromae* LA-SOL3, 1957 genes code for transporters (Table S3).

Heat shock proteins (HSP) are known to be involved in several common biological activities, such as transcription, translation, protein folding, and aggregation and disaggregation of protein, protecting proteins from damages caused by different types of biotic and abiotic stresses^21^. Different families of HSP known to be specifically involved in responses to heat stress were identified in the genome of *L*. *theobromae* (Table S4).

### Transcriptome and proteome of *L*. *theobromae* LA-SOL3 at two temperatures

Temperature deeply influenced the growth (Fig. S1) and both the transcriptome and proteome (Figs S2 and S3).

A total of 1,580 genes were differentially expressed, of which 1,059 genes were annotated, 849 genes were down-regulated, and 731 genes were up-regulated at 37 °C (Fig. S2).

**Figure 2:**
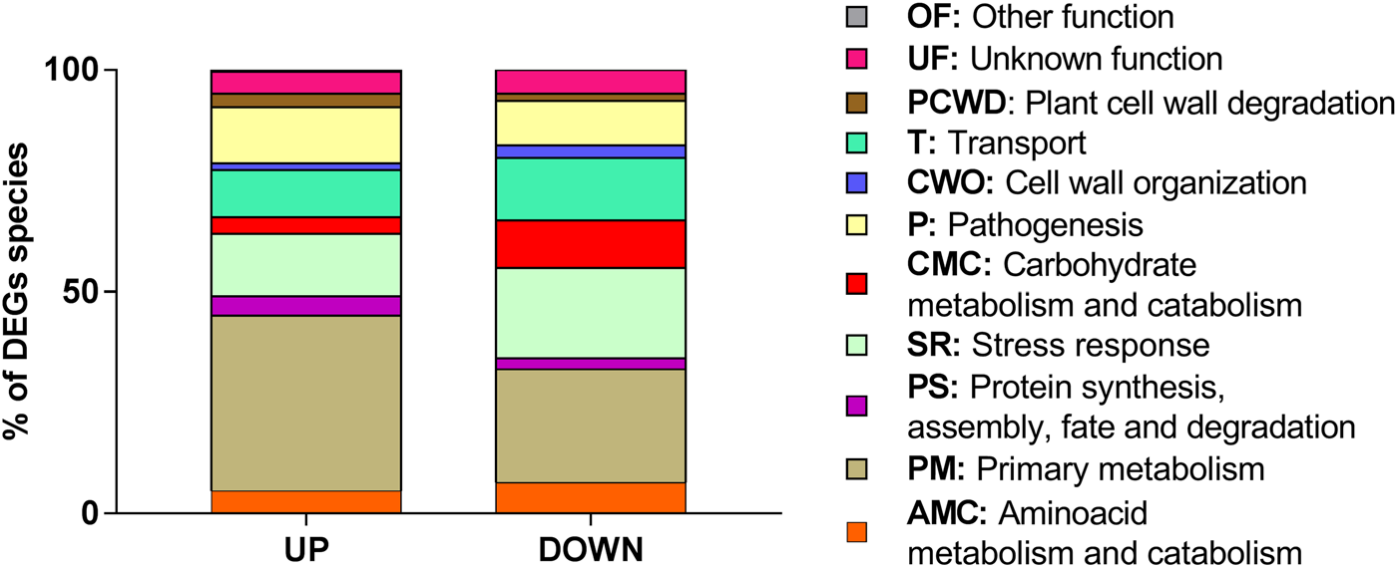
Gene Ontology classification of the transcripts identified in *L*. *theobromae* LA-SOL3: up and down-regulated transcripts of the fungus grown at 37 °C in comparison with transcripts expressed by the fungus grown at 25 °C. The classification was obtained from the GO (biological process) of each gene product according to the UniProt database (http://www.uniprot.org/).

According to GO analysis (Table S9), heat stress leads to an increase of the number of transcripts related to primary metabolism, plant cell wall degradation and pathogenesis, and to a decrease of transcripts related with stress response, carbohydrate metabolism and catabolism and transport functions (Fig. 2).

**Figure 3:**
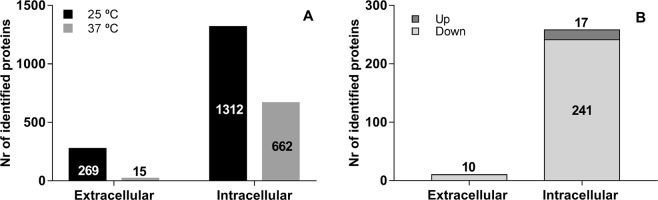
Differential expression of proteins by *L*. *theobromae* LA-SOL3. Total number of identified extracellular and intracellular proteins (**A**) and up and down-regulated proteins expressed under heat stress (**B**). 25 °C corresponds to the control condition.

We identified of 269 proteins in the secretome of *L*. *theobromae* grown at 25 °C and only 15 proteins in the secretome of the fungus grown at 37 °C (15 of which were less abundant than at 25 °C). Regarding the intracellular proteome, a significantly higher number of proteins were identified: 1,312 proteins for 25 °C and 662 for 37 °C (17 more abundant and 241 less abundant than at 25 °C). In both cases, secretome and intracellular proteome, the number of identified proteins were higher when the fungus was grown at the optimal temperature of 25 °C (Fig. 3).

**Figure 4:**
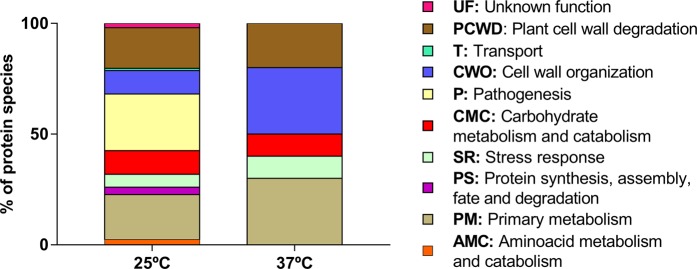
Gene Ontology classification of the extracellular proteins identified in *L*. *theobromae* LA-SOL3: percentage of distinct species present in extracellular proteins at 25 °C and 37 °C. The classification was obtained from the GO (biological process) of each protein according to the UniProt database (http://www.uniprot.org/).

**Figure 5:**
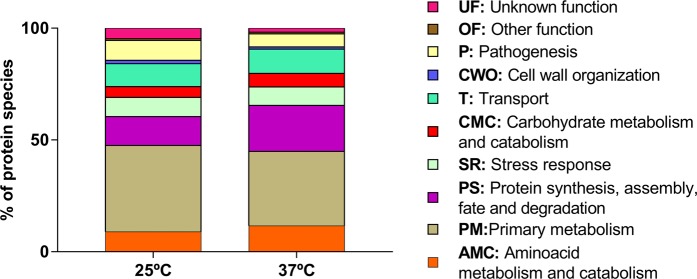
Gene Ontology classification of the intracellular proteins identified in *L*. *theobromae* LA-SOL3: percentage of distinct species present in extracellular proteins at 25 °C and 37 °C. The classification was obtained from the GO (biological process) of each gene product according to the UniProt database (http://www.uniprot.org/).

**Figure 6:**
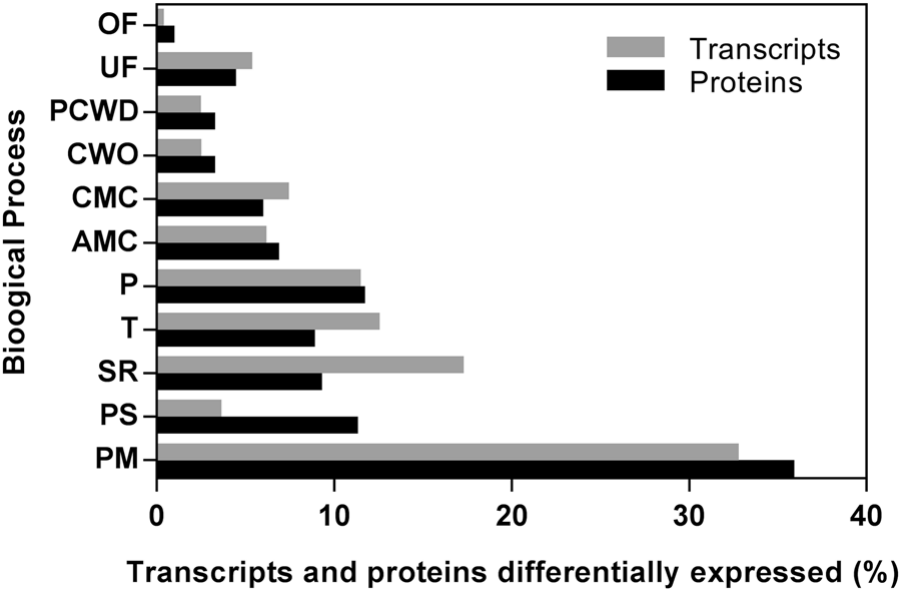
Categorization of *Lasiodiplodia theobromae* differential gene expression and protein levels classified by GO-term (Biological Process) at 25 °C and 37 °C. Proteins detected only at one of the tested temperatures were also included in the analysis. UniProt database was used to access to the GO categories.

When *L*. *theobromae* LA-SOL3 grows at 37 °C, there is a considerable increase of extracellular proteins related to cell wall organisation (CWO), primary metabolism (PM) and stress response (SR). Proteins directly related to pathogenesis (P) were either not detected – in the extracellular compartment (Tables S10 and S11, Fig. 4) – or less abundant intracellularly (Fig. 5). On the other hand, proteins related to protein metabolism were more abundant in the intracellular proteome at 37 °C. The analysis of the GO terms (biological process) of differentially expressed transcripts and proteins shows a similar trend of abundance (Fig. 6).

Some transcripts and proteins previously implicated in for pathogenesis were identified in this study: transcripts/proteins involved in salicylic acid degradation, members of the Velvet complex and mitogen-activated protein kinases (MAPKs). A virulence factor, the virulence protein SSD1 (Q5AK62) was exclusively identified in the intracellular proteome, at 37 °C, while the phytotoxin Snodprot1 (O74238) was identified only in the secretome of LA-SOL3 at 25 °C.

## Discussion

The genome of *L*. *theobromae* LA-SOL3 was compared with that of *L*. *theobromae* CSS-01s (Table 2), a strain that was also isolated from grapevine.

**Table 2:** Genes coding for proteins potentially involved in pathogen-plant interaction in the genome of *L*. *theobromae* LA-SOL3 and comparison with *L*. *theobromae* CSS-01s (Yan *et al*.)^14^.

	LA-SOL3	CSS-01s	Method
Total proteins	12785	12902	BRAKER1
Secreted proteins	677	937	FunSec
Enzymes	4579	—	E2P2
Secreted Enzymes	335	—	FunSec + E2P2
CAZymes	789	763	dbCAN
Secreted CAZymes	272	—	FunSec + dbCAN
Metabolic gene clusters	52	58	fungiSMASH
Cytochrome P450s	44	—	The Cytochrome P450 Homepage
Peroxidases	40	—	fPoxDB
Transporters	1957	2419	Transporter Classification Database
Pathogen-Host Interactions	4075	—	PHI-base

The genome of CSS-01s sequenced by Yan *et al*.^14^ encodes 937 extracellular proteins, accounting for 7.26% of their (predicted) proteome size. In the genome of LA-SOL3, about 5.30% of LA-SOL3 deduced proteome size corresponds to secreted proteins. This difference may be related to the use of the algorithm FunSec. FunSec integrates signal peptides’ and transmembrane regions’, subcellular localisation and endoplasmic reticulum motifs’ prediction. Therefore, FunSec performs a very stringent analysis leading to a reduced number of false positives.

The analysis of the genome *L*. *theobromae* LA-SOL3 is in accordance to the transcripts and proteins identified at 25 and 37 °C: all the functions identified in the genome were also identified in the transcriptome and proteome, although temperature favoured some functions towards others.

From the total amount of genes, 192 genes encode pathogenesis-related genes (GO:0009405). From these 119 were differentially transcribed at 25 °C and 37 °C, and 143 were detected as proteins differentially expressed at 25 and 37 °C (including proteins only detected at one of the temperatures).

At 25 °C (Fig. 5), *L*. *theobromae* LA-SOL3 expresses a large amount of extracellular proteins involved in plant cell wall degradation (18.4%) and in pathogenesis (25.6%), while at 37 °C the percentage of proteins involved in pathogenesis decreased (proteins relevant for pathogenesis are described in Tables 3, S9 and S10). In fact, under stress conditions (37 °C) (Fig. S1), the energy of the fungus was directed to guarantee the survival of the organism, and to the maintenance of the cell wall integrity.

**Table 3:** Functions identified in the transcriptome and proteome of strain LA-SOL3 relevant for pathogenicity.

	Function	Contribution to pathogenicity	Reference
Siderophores	Iron-chelating ligands. Iron transport compounds.	Intracellular iron storage compounds. Suppress the growth of other microorganisms.	Renshaw *et al*.^50^
Toxins	Polyketides that include a range of compounds as mycotoxins and spore pigments.	Toxic to plants and/or animals. Mode of action extremely variable, depending on the produced compound.	Gaffoor *et al*.^51^ Chauhan *et al*.^52^
Allergens	Sensitization with extraneous allergens. Fugal allergens are usually proteins, polysaccharides, or glycoproteins.	IgE-mediated hypersensitivity in humans. The major allergic manifestations are asthma, rhinitis, allergic bronchopulmonary mycoses, hypersensitivity pneumonitis.	Fukutomi & Taniguchi^53^
MAPKs	Mitogen-activated protein kinases that function as key signal transduction components.	Fungal MAPKs help to promote the penetration of host tissues governing appressorium formation and virulence.	Hamel *et al*.^18^ He *et al*.^23^
HSPs	Involved in several common biological activities, such as transcription, translation, protein folding, and aggregation and disaggregation of proteins.	Involved in stress response. Different families of HSP are expressed depending on stress type, *e*.*g*.: pH, heat, cold or osmotic stress.	Tiwari *et al*.^21^
Nudix effectors	Maintenance of proper cellular processes and physiological homeostasis by sensing and modulation levels of their substrates like nucleotide sugars, deoxyribonucleoside triphosphate and capped mRNAs.	Manipulation of host defence systems.	Dong & Wang^54^
Velvet complex	Regulation of fungal development and secondary metabolism.	Promotion of chromatin accessibility and expression of biosynthetic gene clusters involved pathogenicity as mycotoxins, pigments and hormones.	López-Berges *et al*.^55^ Niehaus *et al*.^26^

From those groups of proteins relevant for pathogenesis (Table 3), we highlight the presence of proteins involved in toxins’ synthesis and of MAPKs that are more abundant in the proteome of LA-SOL3 grown at 25 °C.

**Figure 7:**
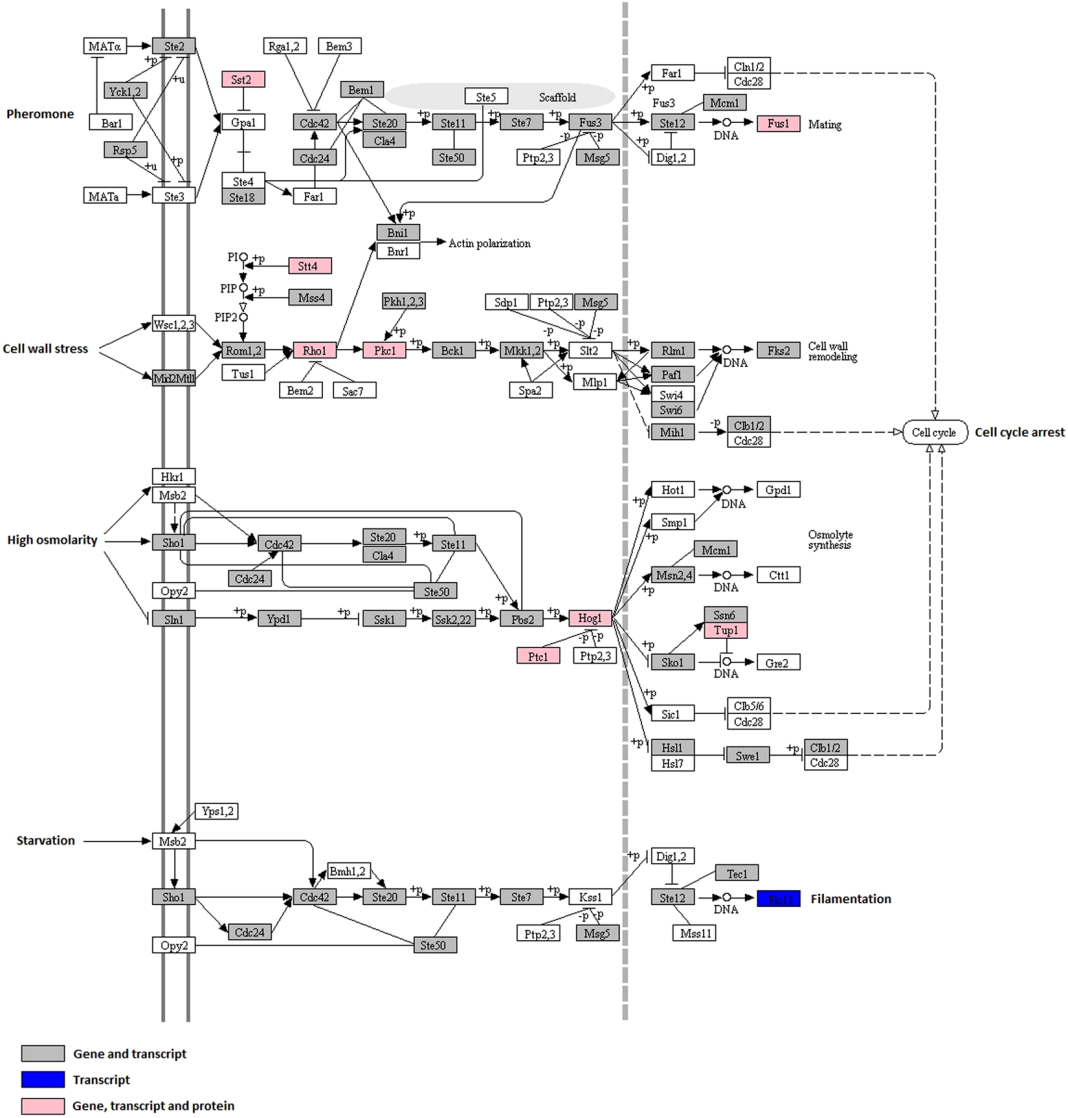
Pathway map assigned for MAPKs signalling. Omics data obtained for LA-SOL3 are highlighted in pink (gene, transcript and protein levels), grey (gene and transcript levels) and blue (transcript level). This figure was adapted from KEGG software, using a reference pathway to construct the map^56–58^.

In the case of plant pathogenic fungi, full virulence requires functional MAPK pathways^18,22,23^. We identified genes, transcripts and proteins essential for the cascades of MAPK pheromone pathway (Fig. 7) (*STE11-STE7-FUS3*, previously identified in *Saccharomyces cerevisiae*, and *PMK1*, previously identified in *Magnaporthe grisea*). We also identified the MAPK genes involved in protection against high osmolarity (*STE11-HOG1*, previously identified in *S*. *cerevisiae* and *OSM1* previously identified in *M*. *grisea*)^22,24,25^. The presence of well-represented pathways of MAPKs signalling that contributes to the successful penetration and dissemination of the fungal pathogen in different hosts, in L. theobromae LA-SOL3 helps to explain how this species is able to infect a wide range of plant hosts and to proliferate in different conditions.

Several HSP genes, transcripts and proteins related to heat response, as is the case of the family clpA/clpB (P31540) and family 70 (P15705), were identified (Table S4). The expression of such molecules allows the pathogen to overcome heat stress since HSP will protect the cells from the possible damages caused by the stress factors^21^.

Transcripts and proteins involved in the Velvet complex were identified in the transcriptome and the intracellular proteome of LA-SOL3 grown at 25 °C. The deletion of one of the proteins of the complex influences the production of specific gene clusters, *e*.*g*., the deletion of *LaeA* gene promotes the decrease of virulence factors’ production in *Fusarium fujikuroi*^26^. Among these virulence factors are the genes and proteins involved in toxin synthesis, such as in the synthesis of fusaric acid and fusarin C, identified in the genome, transcriptome and proteome of *L*. *theobromae*, and in the production of fumonisin (identified in the genome and transcriptome).

The expression of proteins with potential roles in the pathogenesis of *L*. *theobromae* LA-SOL3 (Table 4) was identified.

**Table 4:** Proteins involved in fungi pathogenesis, identified in the proteome of *L*. *theobromae* strain LA-SOL3.

Accession number (UniProtKB)	Description	Secretome	Intracellular Proteome	Function	Reference
P14010	4-aminobutyrate aminotransferase	—	25/37 °C	Metabolization of γ-aminobutyric acid to fulfil pathogen nitrogen requirements during infection and manipulates the plant metabolism to maintain/increase the concentration of nitrogen.	Fernandes^59^
A9MYQ4	Gamma-aminobutyraldehyde dehydrogenase				
Q5AK62	Virulence protein SSD1	—	37 °C	Tolerance of host immune response, allowing the colonization of human tissues by *Candida albicans*.	Gank *et al*.^60^
C5FBW2 Q70J59	Tripeptidyl-peptidase SED2	25 °C	—	Acidification of the microenvironment in the host, facilitating the nutrition and proliferation of the pathogen.	Reichard *et al*.^61^ Félix *et al*.^13^
D4ALG0	LysM domain-containing protein ARB_05157	25 °C	—	Manipulation of host immune responses to support pathogen colonization.	Kombrink & Thomma^45^ Akcapinar *et al*.^62^
O74238	Protein SnodProt1	25 °C	—	Cerato-platanin known to manipulate the immune response and cause necrosis in *Ceratocystis fimbriata*. In *Magnaphorte oryzae* a homologue is required for full virulence.	Brown *et al*.^63^

For all proteins, with the exception of the virulence protein SSD1 and the Snodprot1 protein, that were identified only at the protein level, the expression was confirmed at the mRNA level. SSD1 protein was identified only in the proteome of LA-SOL3 grown at 37 °C. This protein is typically involved in human infections caused by fungi, which helps to explain the capacity of *L*. *theobromae* to infect humans^6^.

Previous studies identified jasmonic acid by *L*. *theobromae* only at 25 °C^27^. In this study, it was possible to identify transcripts (down-regulated) and proteins (only at 25 °C), related to the synthesis of jasmonic acid [e.g. the enzyme 12-oxophytodienoate reductase 7 (Q6Z965)]. Jasmonic acid is a phytotoxin that facilitates the infection process via the inhibition of the plant host defence pathway^28,29^.

We identified other proteins (detected either at the protein or mRNA level) associated with human infections - allergens, aspartic proteases and proteins involved in toxin synthesis (Tables S9 and S10). These data show that *L*. *theobromae* has the molecular machinery to colonise and infect humans and that this machinery is over-expressed when the fungus grows at 37 °C.

Paolinelli-Alfonso *et al*.^17^ showed that carbohydrate catabolism, pectin, starch and sucrose metabolism-related genes, and pentose and glucuronate interconversion pathways were induced during the infection process of grapevine by *L*. *theobromae*^14,17^. The same authors found that enzymes related to plant cell wall degradation were up-regulated at 35 °C. Also, salicylic acid and phenylpropanoid precursors-degrading enzymes were up-regulated, suggesting that evasion from host defence responses could be helped by the L-tyrosine catabolism pathway^17^. In our study, we identified (both by transcriptomics and proteomics) gene products involved in plant cell wall degradation up-regulated at 37 °C. We also identified proteins involved in the degradation of salicylic acid, but at 25 °C. Although data of both studies may seem discordant, a direct comparison between our study and that of Paolinelli-Alfonso *et al*.^17^ is not straightforward or accurate: the presence of the host in the study of Paolinelli-Alfonso *et al*.^17^ leads to multiple factors affecting the expression of proteins by *L*. *theobromae*. In Paolinelli-Alfonso *et al*.^17^ study, not only *L*. *theobromae* was under stress, but the host, *V*. *vinifera*, was also under thermal stress.

Globally, the results obtained show that *L*. *theobromae* has not only the ability to behave like a typical phytopathogen, expressing molecules involved in the plant cell wall or salicylic acid degradation but also has the necessary toolbox to infect mammals, expressing proteins typically involved in the infection of humans. Several proteins typically involved in human pathogenesis are expressed only at 37 °C, suggesting that the pathogenicity of *L*. *theobromae* is modulated by temperature.

Moreover, the presence of genes and proteins known to be involved not only in necrosis of host tissues (genes/proteins involved in synthesis of toxins or virulence factors as the snodprot protein) but also in mechanisms that allow the pathogen to persist inside a living host (as the degradation of salicylic acid or the expression of the protein SSD1), suggest that this species may have a hemibiotrophic lifestyle.

Having in mind the ongoing climate changes, one may speculate that an increase in temperature although leading to heat stress of *L*. *theobromae*, may also lead to an increase on the severity of human infections by this opportunist pathogen.

## Methods

### Fungal strains and culture conditions

The strain used in this study was *L*. *theobromae* LA-SOL3, isolated from *Vitis vinifera* in Peru. LA-SOL3 was one of the most aggressive strains in artificial inoculations trials of cv. Red Globe plants^2^. The culture was routinely grown on Potato Dextrose Agar (PDA) medium (Merck, Germany) at 25 °C.

For DNA extraction, the mycelium harvested from a culture grown on Potato Dextrose Broth (PDB, Merck, Germany) at 25 °C for three days was ground in liquid nitrogen and DNA was extracted according to Möller *et al*.^30^. For protein and RNA extraction, LA-SOL3 was inoculated into a 250 mL flask containing 50 mL of PDB and incubated at 25 °C or at 37 °C for 4 days, as described earlier^31^. All assays were performed in triplicate.

### DNA extraction, Genome sequencing and assembly

*Lasiodiplodia theobromae* strain LA-SOL3 was sequenced from 500 ng of genomic DNA by Illumina MiSeq at NXTGNT (Belgium). The DNA was fragmented to 800 bp using Covaris S2 sonication, and a sequence library was made using the NEBNext Ultra DNA Library Prep Kit (New England BioLabs, Ipswich, MA, USA). During the library preparation, a size selection was performed just before the enrichment PCR using an Invitrogen 1% E-gel to select for fragments between 700 and 1200 bp. Nine PCR cycles were used during the enrichment PCR. The libraries were equimolarly pooled and sequenced on an Illumina MiSeq sequencer, generating about 19E + 06 2 × 300 bp paired-end reads. Base calling and primary quality assessments were performed using Illumina’s Basespace genomics cloud computing environment. Reads were trimmed based on quality scores (modified Mott trimming algorithm, threshold = 0.05) and reads shorter than 100 nt were discarded. Detected adaptor sequences were also trimmed and reads mapping to the Illumina internal control phage phiX were discarded. Assembly and scaffolding were achieved with CLC Genomics Workbench 9.0.1 (https://www.qiagenbioinformatics.com/) *de novo* assembly module with default settings, except for the minimum contig length (2000 nt) or word length (60 nt). Optimal word size was estimated using KmerGenie28^32^ prior to the assembly. The primary scaffolds were further refined by means of the SSPACE2 scaffolder. The gapped regions in the re-scaffolded assembly were (partially) closed using GapFiller^33^.

### Gene prediction and annotation

Before gene prediction, the genome was repeat-masked. RepeatModeler (v1.0.8)^34^ was used to build repeat libraries for the *L*. *theobromae* genome, which were then filtered to exclude coding regions and simple repeats. RepeatMasker (v4.0.5) was used to “hard mask” repeats and transposable elements in the genomes, using the curated repeat libraries, replacing repeats and transposable elements by strings of ‘N’s.

Gene prediction was achieved with BRAKER1 (v1) with the “fungus” option. RNA-seq paired-end reads from the GEO study GSE75978^17^ were selected (SRR2994047, SRR2994048, SRR2994053, SEE2994054, SRR2994059, SRR2994060, SRR2994062 and SRR2994063) and used for mapping onto the assembled genome using HISAT2 (v2.0.5)^35^ with default settings.

Gene product names were assigned based on predicted protein sequences with BLASTP against the UniProt-KB/SwissProt database (downloaded on March 1^st^, 2017), with an E-value threshold of 1e-3. Other functional annotations were added with InterProScan (v5.21-60)^36^, mapping accessions for InterPro, GO, CDD-3.14 and Pfam-30.0 as well as GO terms. Further functional analyses were carried out as described by Morales-Cruz *et al*.^37^.

### Genome functional analysis

Secreted gene products were predicted with the FunSec pipeline (v1.0) (https://github.com/Lonewolfenrir/FunSec). InterProScan analysis and the Ensemble Enzyme Prediction Pipeline (E2P2) were used to annotate protein sequences. The results were divided into different categories based on their EC numbers.

Carbohydrate-degrading enzymes (CAZymes) were predicted with the web-based application dbCAN (HMMs 5.0) with default settings^38^. Fungal peroxidases were predicted with the web-based BLAST application of fPoxDB: Fungal Peroxidase Database^39^, against the Whole Predicted Peroxidases database (updated on April 18^th^, 2012), with an E-value threshold of 1e^−5^. Gene clusters encoding for secondary metabolites were predicted for the full genomes with the web-based application fungiSMASH (antiSMASH fungal version v4)^40^, with added GFF3-formatted files, obtained from gene prediction, and ran with default settings. Cytochrome P450 identification was pursued by BLAST against The Cytochrome P450 Homepage^41^ database, with an E-value threshold of 1e^−5^, against the fungal database (updated on August 12^th^, 2009). A BLAST analysis was conducted against the Pathogen-Host Interactions database (PHI-base), downloaded on February 2^nd^, 2017, against the whole proteome and the secreted protein datasets, with an E-value threshold of 1e-5. Transporters were identified with a BLAST analysis against the Transporter Classification Database^42^, downloaded on January 14^th^, 2018, using an E-value threshold of 1e^−5^.

### RNA extraction, library preparation and sequencing

The mycelium of each replicate (three replicates per condition) was ground in liquid nitrogen, and total RNA was extracted using the Spectrum Plant Total RNA kit (Sigma), according to the manufacturer’s instructions. Samples were incubated for 15 min with the DNase I digestion set (RNase-Free DNase Set, Qiagen). Integrity and quality analysis were done on a 2100 Bioanalyzer RNA (Agilent Technologies). Afterwards, samples were stored at −80 °C until sequencing library preparation. Illumina mRNA sequencing libraries were made from 500 ng total RNA of each sample using the QuantSeq. 3′ mRNA-Seq Library Prep Kit (Lexogen, Vienna, Austria) according to the manufacturer’s protocol. Fourteen PCR cycles were used during the enrichment PCR. The size distribution, purity (absence of free adaptors) and quantity of the resulting libraries were measured using a High Sensitivity DNA chip (Agilent Technologies, Santa Clara, CA, US). The libraries were equimolarly pooled and sequenced in an Illumina Nextseq. 500 high throughput flow cell, generating single-end 75 bp reads. After sequencing, the data was demultiplexed using the sample-specific nucleotide barcodes. Per sample, on average, 38 × 10^6^ ± 4 × 10^6^ reads were generated. First, these reads were trimmed using cutadapt version 1.11. To remove the “QuantSEQ FWD” adaptor sequences, the trimmed reads were mapped to the genome of *L*. *theobromae* LA-SOL3 using the STAR aligning software v 2.5.3a^43^. The RSEM software, version v1.2.31, was used to generate count tables for *L*. *theobromae*. Sequencing and mapping quality reports were aggregated with MultiQC version 1.0. Raw reads and raw count data are accessible in the Gene Expression Omnibus (GSE131341).

### Proteome analysis

The extracellular fraction of the proteome was obtained with the TCA/Acetone method^44^. The final pellet was resuspended with 200 µL of lysis buffer (7 M urea, 2 M thiourea, 4% CHAPS, 30 mM Tris) and stored at −80 °C.

For the intracellular fraction of the proteome, mycelia were grinded to a fine powder in a mortar in N_2_(l). To the powder, 10 mL of 10 mM potassium-phosphate buffer (pH 7.4) containing 0.07% DTT and Complete™ protease inhibitor cocktail (Roche) was added. All the samples were sonicated [1 min sonication, 2 min pause (3 min of sonication in total) (Branson, Sonifier 250) at 4 °C] and then shaken using an orbital agitator at minimum speed, during 2 h (4 °C). The homogenate was centrifuged (15000 g, 30 min, 4 °C), and the supernatant was collected. Proteins from the extract were precipitated following the same procedure used to extract extracellular proteins.

Protein concentration was determined with the 2-D Quant Kit (GE Healthcare, USA), according to the manufacturer’s instructions. All the samples were quantified in triplicate. For both intra- and extracellular proteins, 125 µg of the sample was diluted (1:1) in loading buffer and analysed by electrophoresis^45^. Lab-casted SDS-PAGE gels ran at 2 W-2 h, 6 W-3 h on 12% (w/v). The running buffer contained 100 mM Tris, 100 mM Bicine and 0.1% (w/v) SDS^13^. The samples were denatured at 100 °C for 5 min prior to electrophoresis^13^. Gels were stained with Coomassie Brilliant Blue G-250 (CBB), as described earlier^43^. After staining, gels were scanned on a GS-800 Calibrated Densitometer (Bio-Rad).

After ensuring the quality of the samples, these were electrophoresed and concentrated at the top of the separation gel (visualised as a unique band). This band was manually excised, and protein bands were treated as described by Padilla and colleagues^46^ with slightly modifications^47^. Bands were destained in 200 mM ammonium bicarbonate (AB)/50% acetonitrile for 15 min and 5 min in 100% acetonitrile. Proteins were reduced by the addition of 20 mM dithiothreitol in 25 mM AB and incubated for 20 min at 55 °C. The mixture was cooled to room temperature, followed by alkylation of free thiols by the addition of 40 mM iodoacetamide in 25 mM AB in the dark, for 20 min. After that, protein bands were washed twice in 25 mM AB. Proteolytic digestion was performed by adding 12.5 ng.µL^−1^ of trypsin (Promega, Madison, WI) in 25 mM AB, and incubated at 37 °C overnight. Protein digestion was stopped by addition of trifluoroacetic acid (TFA) at 1% final concentration. Digested samples were dried in a speedvac.

A nano LC analysis was performed according to Padilla *et al*.^46^, with slight modifications^47^, using a Dionex Ultimate 3000 nano UPLC (Thermo Scientific) with a C18 75 μm × 50 Acclaim Pepmam column (Thermo Scientific). Previously, the peptide mix was loaded in a 300 μm × 5 mm Acclaim Pepmap precolumn (Thermo Scientific) in 2% acetonitrile/0.05% TFA for 5 min at 5 µL.min^−1^. Peptide separation was performed at 40 °C. Mobile phase A was composed of water acidified with 0.1% formic acid. Mobile phase B was composed of 20% acetonitrile acidified with 0.1% formic acid. Samples were separated at 300 nL.min^−1^. Mobile phase B increased from 4% to 45% B in 60 min; 45–90% B in 1 min, followed by a 5 min wash at 90% B and a 15 min re-equilibration at 4% B. The total time of chromatography was 85 min.

Eluting peptide cations were converted to gas-phase ions by nano-electrospray ionisation and analysed in a Thermo Orbitrap Fusion (Q-OT-qIT, Thermo Scientific). The mass spectrometer was operated in positive mode. Survey scans of peptide precursors from 400 to 1,500 m/z were performed at 120 K resolution (at 200 m/z) with a 5 × 10^5^ ion count target. Tandem MS was performed by isolation at 1 Th with the quadrupole, CID fragmentation with a normalised collision energy of 35, and rapid scan MS analysis in the ion trap. The AGC ion count target was set to 10^2^, and the max injection time was 75 ms. Only those precursors with charge state 2–6 were sampled for MS2. The dynamic exclusion duration was set to 15 s with a 10 ppm tolerance around the selected precursor and its isotopes. Monoisotopic precursor selection was turned on. The instrument was run in top speed mode with 3 s cycles, meaning the instrument would continuously perform MS2 events until the list of non-excluded precursors diminishes to zero or 3 s, whichever is shorter.

The raw data were processed according to Cuevas-Fernández *et al*.^48^, using Proteome Discoverer (version 2.1.0.81, Thermo Scientific). MS2 spectra were searched with SEQUEST engine against an in-house built database of proteins deduced from the genomic sequence. Peptides were generated from a tryptic digestion with up to one missed cleavage, carbamidomethylation of cysteines as fixed modifications, and oxidation of methionines as variable modifications. Precursor mass tolerance was 10 ppm, and productions were searched at 0 Da tolerance.

### Statistical analysis

For transcriptomic analysis, raw reads were normalised using edgeR standard normalisation method^49^. Subsequently, genes were filtered: only genes with a counts per million (cpm) above 1 in at least three samples were retained for statistical differential gene expression analysis. Differential gene expression analysis was performed using EdgeR^49^. A general linear model was built, and statistical testing was done using the empirical Bayes quasi-likelihood (QL) F-test, adjusted using the Benjamin-Hochberg False Discovery Rate (FDR) correction accounting for multiple comparisons. Genes having a Fold Change (log2 FC > 1 or < −1) and a False Discovery Rate (FDR < 0.01) were considered significantly differentially expressed genes (DEGs). To understand the functions of the DEGs a Gene Ontology (GO) analysis was performed using the GOslim software.

For proteomics analysis, the peptide spectral matches (PSM) were validated using percolator based on q-values at a 1% FDR. With Proteome Discoverer software v. 2.1 (Thermo Scientific), peptide identifications were grouped into proteins according to the law of parsimony and filtered to 1% FDR. The identified proteins were also filtered and considered for analysis only if present in three replicates and using at least three peptides for identification (Tables S5–S8). The abundance level was obtained considering the temperature of 25 °C as control (0.5 ≥ FC ≥ 2).

## Supplementary information

Supplementary Information

Supplementary Information

Supplementary dataset

## Data Availability

The datasets generated during and analysed during the current study are available in the GenBank repository (SUB3910116) and the Gene Expression Omnibus (GSE131341). Data generated or analysed during this study are included in this published article [and its supplementary information files].
