# Supplementary material for: A multi-omics analysis of the grapevine pathogen Lasiodiplodia theobromae reveals that temperature affects the expression of virulence- and pathogenicity-related genes
Source: Sci Rep. 2019 Sep 11;9:13144. doi: 10.1038/s41598-019-49551-w (PMC6739476; doi:10.1038/s41598-019-49551-w)
Supplement: Supplementary file 1 — Supplementary Information [file 41598_2019_49551_MOESM1_ESM.docx]

**A multi-omics analysis of the grapevine pathogen *Lasiodiplodia theobromae* reveals that temperature affects the expression of virulence- and pathogenicity-related genes**

Carina Félix^1^, Rodrigo Meneses^1,2^, Micael F. M. Gonçalves^1^, Laurentijn Tilleman^3^, Ana S. Duarte^1^, Jesus V. Jorrín-Novo^4^, Yves Van de Peer^2^, Dieter Deforce^3^, Filip Van Nieuwerburgh^3^, Ana C. Esteves^1, a^, Artur Alves^1,*^

**Figure S1** Radial growth of *L. theobromae* LA-SOL3 after 48 h of incubation in PDB medium at different temperatures. Data is presented as average ± standard error. Two-way ANOVA, followed by a Bonferroni multiple comparison test, was used to determine the statistical significance between the temperature of 30 ºC and all the other temperatures (*p <0.05, **p < 0.01, ***p < 0.001, ****p < 0.0001).

**
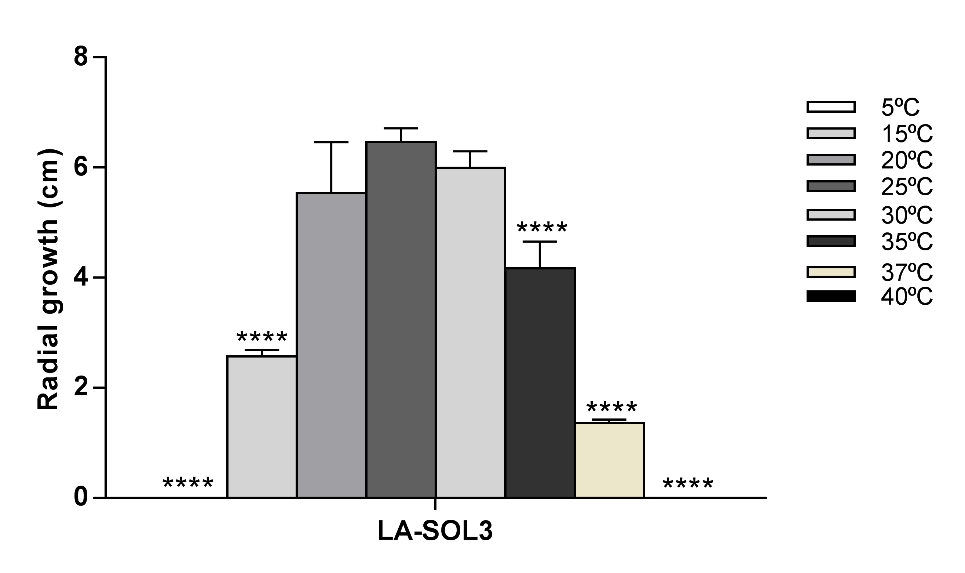
**

**Figure S2** Volcano plot and hierarchical cluster analysis of the expression profiles of differentially expressed genes between 25 °C and 37 °C.

**
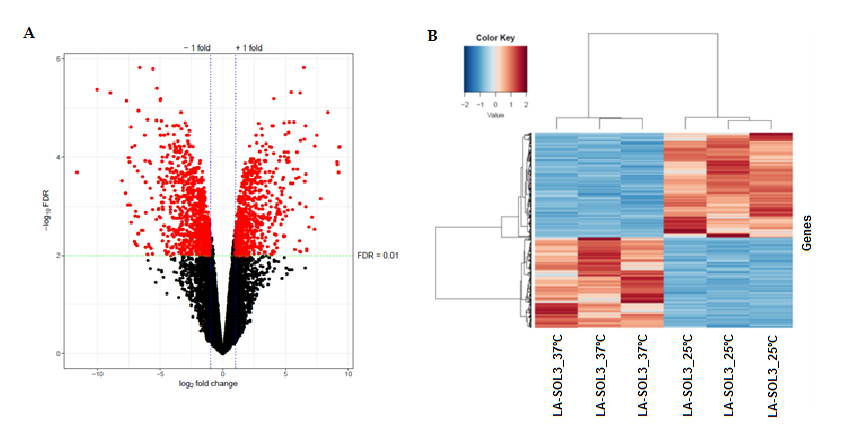
**

**Figure S3** One-DE image of extracellular medium and mycelium of LA-SOL3 strain grown at 25 °C and 37 °C for 4 days, obtained from One-DE gels.

**
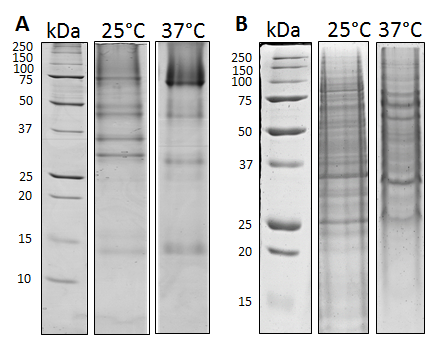
**

**Table S1** Genes predicted to code for fungal peroxidases in the genome of *L. theobromae* LA-SOL3. Peroxidases were predicted with the web-based BLAST application of fPoxDB: Fungal Peroxidase Database.

| **Peroxidase** | **Genes (n)** |
| --- | --- |
| New NoxA | 6 |
| Other class II peroxidase | 6 |
| Haloperoxidase (haem) | 5 |
| Linoleate diol synthase (PGHS like) | 5 |
| New NoxC | 4 |
| Atypical 2-Cysteine peroxiredoxin (type Q, BCP) | 4 |
| Cytochrome C peroxidase | 4 |
| Manganese peroxidase | 4 |
| New NoxB | 4 |
| 1-Cysteine peroxiredoxin | 3 |
| Catalase | 3 |
| Hybrid Ascorbate-Cytochrome C peroxidase | 3 |
| Lignin peroxidase | 3 |
| Typical 2-Cysteine peroxiredoxin | 3 |
| Versatileperoxidase | 3 |
| Atypical 2-Cysteine peroxiredoxin (type II, type V) | 2 |
| Carboxymuconolactone decarboxylase  (no peroxidase activity) | 2 |
| Prostaglandin H synthase (Cyclooxygenase) | 1 |
| New Rbohs | 1 |
| Fungi-Bacteria glutathione peroxidase | 1 |
| NoxR | 1 |
| New_Doux | 0 |
| DyP-type peroxidase D | 0 |
| No haem, Vanadium chloroperoxidase | 0 |

**Table S2** Fungal cytochrome P450 families predicted to be encoded by *L. theobromae* LA-SOL3.

| **Family** | **Genes (n)** |
| --- | --- |
| CYP504 | 13 |
| CYP505 | 2 |
| CYP5080 | 1 |
| CYP531 | 1 |
| CYP532 | 2 |
| CYP545 | 1 |
| CYP547 | 1 |
| CYP548 | 1 |
| CYP56 | 3 |
| CYP570 | 1 |
| CYP573 | 1 |
| CYP578 | 2 |
| CYP586 | 1 |
| CYP596 | 1 |
| CYP6001 | 1 |
| CYP617 | 1 |
| CYP620 | 1 |
| CYP626 | 2 |
| CYP633 | 1 |
| CYP65 | 1 |
| CYP664 | 1 |
| CYP671 | 2 |
| CYP682 | 2 |

**Table S3** Genes predicted to code for transporters in the genome of *L. theobromae* LA-SOL3.

| **Transporter Class** | **Genes (n)** |
| --- | --- |
| Channels and pores | 166 |
| Electrochemical potential-driven transporters | 857 |
| Primary active transporters | 737 |
| Accessory factors involved in transport | 44 |
| Transmembrane electron carriers | 29 |
| Group translocators | 20 |
| Incompletely characterized transport systems | 104 |
| **Total** | **1957** |

**Table S4** Predicted heat shock proteins involved in responses to heat stress in the genome of *L. theobromae* LA-SOL3.

| **HSP name** | **HSP Family** | **Accession Code** |  |
| --- | --- | --- | --- |
| Heat shock protein STI1 | HSP 70 | P15705 |  |
| 30 kDa heat shock protein | HSP 30 | P40920 |  |
| Heat shock protein hsp98 | HSP clpA/clpB | P31540 |  |
| 12 kDa heat shock protein | HSP 20 | P22943 |  |
| Heat shock protein 78, mitochondrial | HSP clpA/clpB | Q96UX5 |  |
| Heat shock protein 16 | HSP20 | O14368 |  |

**Table S5** Proteins identified in the secretome of LA-SOL3 strain at 25 °C.

**Table S6** Proteins identified in the intracellular proteome of LA-SOL3 strain at 25 °C.

**Table S7** Proteins identified in the secretome of LA-SOL3 strain at 37 °C.

**Table S8** Proteins identified in the intracellular proteome of LA-SOL3 strain at 37 °C.

**Table S9** Up and down-regulated genes at 37 °C identified in LA-SOL3 strain and correspondent GO biological process.

**Table S10** Up and down-regulated proteins (0.5 ≥ FC ≥ 2) identified in the secretome of LA-SOL3 strain and correspondent GO biological process.

**Table S11** Up and down-regulated proteins (0.5 ≥ FC ≥ 2) identified in the intracellular proteome of LA-SOL3 strain and correspondent GO biological process.

**Table S12** Potentially relevant proteins identified in the secretome of strain LA-SOL3 grown at 25 °C and 37 °C.

| **GO Category** | **Accession code** | **Description** | **Gene names** | **Temperature (°C)** |
| --- | --- | --- | --- | --- |
| **Pathogenesis** | B8NM69 | Peptidase S41 family protein ustP (EC 3.4.-.-) | ustP AFLA_095010 | 25 |
|  | C5FBW2 | Tripeptidyl-peptidase SED2 (EC 3.4.14.10) | SED2 MCYG_00184 | 25 |
|  | C5FW30 | Carboxypeptidase S1 homolog A (EC 3.4.16.6) | SCPA MCYG_06933 | 25 |
|  | C9SPU8 | Probable zinc metalloprotease VDBG_06923 (EC 3.4.-.-) | VDBG_06923 | 25 |
|  | D4ALG0 | LysM domain-containing protein ARB_05157 | ARB_05157 | 25 |
|  | D4ALQ5 | Probable extracellular glycosidase ARB_05253 (EC 3.2.-.-) | ARB_05253 | 25 |
|  | D4AP52 | Carboxypeptidase S1 homolog B (EC 3.4.16.6) | SCPB ARB_06019 | 25 |
|  | D4APQ6 | Probable thioredoxin reductase ARB_06224 (EC 1.8.1.9) | ARB_06224 | 25 |
|  | D4AQA7 | Probable serine carboxypeptidase ARB_06414 (EC 3.4.16.-) | ARB_06414 | 25 |
|  | D4ARB1 | Probable dipeptidyl-peptidase 5 (EC 3.4.14.-) | DPP5 ARB_06651 | 25 |
|  | D4AYB4 | Antigenic thaumatin-like protein ARB_01183 | ARB_01183 | 25 |
|  | D4AYS6 | Probable extracellular serine carboxypeptidase (EC 3.4.-.-) | ARB_01345 | 25 |
|  | D4B327 | Probable pathogenesis-related protein ARB_02861 | ARB_02861 | 25 |
|  | D4B5N0 | Metallocarboxypeptidase A-like protein ARB_03789 (EC 3.4.17.-) | ARB_03789 | 25 |
|  | D7UQ40 | Bifunctional solanapyrone synthase (EC 1.1.3.42) (Prosolanapyrone-II oxidase) | sol5 | 25 |
|  | G3XMD0 | FAD-linked oxidoreductase azaL (EC 1.-.-.-) | azaL ASPNIDRAFT_132654 | 25 |
|  | M1W428 | Thioredoxin reductase tcpT (EC 1.8.1.-) | tcpT CPUR_02681 | 25 |
|  | O42630 | Vacuolar protease A (EC 3.4.23.25) | pep2 AFUA_3G11400 | 25 |
|  | O42799 | Allergen Asp f 7 | AFUA_4G06670 | 25 |
|  | O64411 | Polyamine oxidase (EC 1.5.3.14) (EC 1.5.3.15) | PAO | 25 |
|  | O74238 | Protein SnodProt1 | SNOG_13722 | 25 |
|  | P11838 | Endothiapepsin (EC 3.4.23.22) | EAPA EPN-1 | 25 |
|  | P24665 | Aspergillopepsin-2 (EC 3.4.23.19) |  | 25 |
|  | P29717 | Glucan 1,3-beta-glucosidase (EC 2.4.1.-) (EC 3.2.1.58) | XOG1 EXG EXG1 XOG CAALFM_C102990CA Ca49C10.05 | 25 |
|  | P34946 | Carboxypeptidase S1 (EC 3.4.16.6) |  | 25/37 |
|  | P36196 | Acetylcholinesterase (EC 3.1.1.7) | ACHE | 25 |
|  | P39105 | Lysophospholipase 1 (EC 3.1.1.5) | PLB1 YMR008C YM8270.10C | 25 |
|  | P42893 | Endothelin-converting enzyme 1 (EC 3.4.24.71) | Ece1 | 25 |
|  | P52719 | Carboxypeptidase cpdS (EC 3.4.16.-) | cpdS | 25 |
|  | P58099 | C5a peptidase (EC 3.4.21.110) | scpA SPy_2010 M5005_Spy1715 | 25 |
|  | P64744 | Sphingomyelinase (EC 3.1.4.12) | BQ2027_MB0912 | 25 |
|  | P79085 | Major allergen Alt a 1 | ALTA1 | 25 |
|  | P86325 | Carboxylesterase (EC 3.1.1.1) |  | 25 |
|  | Q00668 | Putative sterigmatocystin biosynthesis peroxidase stcC (EC 1.11.1.-) | stcC AN7823 | 25 |
|  | Q03168 | Lysosomal aspartic protease (EC 3.4.23.-) | AAEL006169 | 25 |
|  | Q0D1P3 | Multicopper oxidase terE (EC 1.-.-.-) | terE ATEG_00141 | 25 |
|  | Q0V1D7 | Neutral protease 2 homolog SNOG_02177 (EC 3.4.24.39) | SNOG_02177 | 25 |
|  | Q4WFX9 | Probable leucine aminopeptidase 2 (EC 3.4.11.-) | lap2 AFUA_3G00650 | 25 |
|  | Q4WNV0 | Aspartic-type endopeptidase ctsD (EC 3.4.23.-) | ctsD AFUA_4G07040 | 25 |
|  | Q4WZS3 | Putative aspergillopepsin A-like aspartic endopeptidase AFUA_2G15950 (EC 3.4.23.-) | AFUA_2G15950 | 25 |
|  | Q5AZ42 | Probable dipeptidyl peptidase 4 (EC 3.4.14.5) | dpp4 AN6438 | 25 |
|  | Q70J59 | Tripeptidyl-peptidase sed2 (EC 3.4.14.10) | sed2 sedB AFUA_4G03490 | 25 |
|  | Q871C5 | Extracellular metalloprotease NCU07200 (EC 3.4.24.-) | B8G12.220 NCU07200 | 25 |
|  | Q8RJP2 | Rhamnogalacturonate lyase (EC 4.2.2.23) | rhiE Dda3937_01465 | 25 |
|  | Q92396 | Tyrosinase (EC 1.14.18.1) | TYR | 25 |
|  | Q9DDE3 | Acetylcholinesterase (EC 3.1.1.7) | ache | 25 |
|  | Q9Y7F0 | Peroxiredoxin TSA1-A (EC 1.11.1.15) | TSA1 TSA1A CAALFM_C306180CA CaO19.7417 | 25 |
| **Stress Response** | B0Y004 | Cell wall protein phiA (Major allergen phiA) | phiA AFUB_045170 | 25 |
|  | B9W4V6 | Aromatic peroxygenase (EC 1.11.2.1) | APO1 | 25 |
|  | C0IW58 | Low-redox potential peroxidase (EC 1.11.1.7) | LnP | 25 |
|  | D4AUF1 | WSC domain-containing protein ARB_07867 | ARB_07867 | 25 |
|  | D4AUF4 | WSC domain-containing protein ARB_07870 | ARB_07870 | 25 |
|  | G0Y276 | Effector protein PevD1 |  | 25 |
|  | P29141 | Minor extracellular protease vpr (EC 3.4.21.-) | vpr BSU38090 ipa-45r | 25 |
|  | Q3HRQ2 | Aldehyde oxidase GLOX (EC 1.2.3.1 | GLOX | 25 |
|  | Q4WNS8 | Protein ecm33 | ecm33 AFUA_4G06820 | 25 |
|  | Q4WXZ5 | Ribonuclease T2-like (EC 3.1.27.1) | rny1 AFUA_3G11220 | 25 |
|  | Q86WA6 | Valacyclovir hydrolase (EC 3.1.-.-) | BPHL MCNAA | 25 |
|  | Q877A8 | Catalase B (EC 1.11.1.6) | catB AO090120000068 | 25 |
|  | B5Y008 | Oxygen-dependent choline dehydrogenase (EC 1.1.99.1) (EC 1.2.1.8) | betA KPK_3995 | 37 |
| **Cell Wall Degradation** | A1C4H2 | Probable endo-beta-1,4-glucanase D (EC 3.2.1.4) | eglD ACLA_059790 | 25 |
|  | A1DBG6 | Probable beta-glucosidase btgE (EC 3.2.1.21) | btgE NFIA_098360 | 25 |
|  | A1DBS6 | Probable endo-beta-1,4-glucanase D (EC 3.2.1.4) | eglD NFIA_099510 | 25 |
|  | A1DME8 | Probable endo-beta-1,4-glucanase B (EC 3.2.1.4) | eglB NFIA_053150 | 25 |
|  | A1DMR8 | Probable beta-glucosidase F (EC 3.2.1.21) | bglF NFIA_054350 | 25/37 |
|  | A1DMV3 | Probable feruloyl esterase B-2 (EC 3.1.1.73) | faeB-2 NFIA_054700 | 25 |
|  | A2QT85 | Probable arabinan endo-1,5-alpha-L-arabinosidase A (EC 3.2.1.99) | abnA An09g01190 | 25 |
|  | A2QYR9 | Probable 1,4-beta-D-glucan cellobiohydrolase C (EC 3.2.1.91) | cbhC An12g02220 | 25 |
|  | B8MW97 | probable endo-beta-1,4-glucanase B (EC 3.2.1.4) | eglB AFLA_087870 | 25 |
|  | B8MXJ7 | Probable endo-beta-1,4-glucanase D (EC 3.2.1.4) | eglD AFLA_077840 | 25 |
|  | B8NJF4 | Probable beta-glucosidase D (EC 3.2.1.21) | bglD AFLA_066750 | 25 |
|  | B8NMD3 | Probable arabinan endo-1,5-alpha-L-arabinosidase C (EC 3.2.1.99) | abnC AFLA_123690 | 25 |
|  | B8NPT0 | Probable feruloyl esterase B-2 (EC 3.1.1.73) | faeB-2 AFLA_001440 | 25 |
|  | D4AJR9 | Endo-1,3(4)-beta-glucanase ARB_04519 (EC 3.2.1.6) | ARB_04519 | 25 |
|  | D4AV38 | Probable secreted lipase ARB_00047 (EC 3.1.1.1) | ARB_00047 | 25 |
|  | D4AZ24 | Probable endo-1,3(4)-beta-glucanase ARB_01444 ( (EC 3.2.1.6) | ARB_01444 | 25 |
|  | D4AZ78 | Secreted lipase ARB_01498 (EC 3.1.1.3) | ARB_01498 | 25 |
|  | P07982 | Endoglucanase EG-II (EC 3.2.1.4) | egl2 | 25 |
|  | P0C1A6 | Pectate lyase L (EC 4.2.2.2) | pelL | 25 |
|  | P23360 | Endo-1,4-beta-xylanase (EC 3.2.1.8) | XYNA | 25/37 |
|  | P23550 | Endoglucanase B (EC 3.2.1.4) | celB | 25 |
|  | P41365 | Lipase B (EC 3.1.1.3) (CALB) |  | 25 |
|  | P43317 | Endoglucanase-5 (EC 3.2.1.4) | egl5 | 25 |
|  | P45699 | Putative endoglucanase type K (EC 3.2.1.4) |  | 25 |
|  | P53626 | Glucan endo-1,3-beta-glucosidase BGN13.1 (EC 3.2.1.39) | bgn13.1 | 25 |
|  | P55332 | Endo-1,4-beta-xylanase A (EC 3.2.1.8) | xlnA AN3613 | 25 |
|  | Q01738 | Cellobiose dehydrogenase (EC 1.1.99.18) | CDH-1; CDH-2 | 25 |
|  | Q0CEF3 | Probable beta-glucosidase L (EC 3.2.1.21) | bglL ATEG_07931 | 25 |
|  | Q0CMT2 | Probable 1,4-beta-D-glucan cellobiohydrolase B (EC 3.2.1.91) | cbhB ATEG_05002 | 25 |
|  | Q0CTD7 | Probable beta-glucosidase A (EC 3.2.1.21) | bglA bgl1 ATEG_03047 | 25 |
|  | Q2U7D2 | Probable alpha-L-arabinofuranosidase axhA (EC 3.2.1.55) | axhA AO090701000885 | 25 |
|  | Q45071 | Arabinoxylan arabinofuranohydrolase (EC 3.2.1.55) | xynD BSU18160 | 25 |
|  | Q5B9F2 | Probable beta-glucosidase L (EC 3.2.1.21) | bglL AN2828 | 25 |
|  | Q5ZNB1 | Endo-1,4-beta-xylanase D (Xylanase D) (EC 3.2.1.8) | xynD | 25 |
|  | Q92194 | Acetylxylan esterase A (EC 3.1.1.72) | axeA aceA | 25 |
|  | Q96VB6 | Endo-1,4-beta-xylanase F3 (EC 3.2.1.8) | xynF3 xlnF3 AO090001000208 | 25 |
|  | Q96WQ9 | Probable endo-beta-1,4-glucanase D (EC 3.2.1.4) | eglD cel61A AKAW_08531 | 25 |
|  | Q99034 | Acetylxylan esterase (EC 3.1.1.72) | axe1 | 25 |

**Table S13** Potentially relevant proteins identified in the intracellular proteome of strain LA-SOL3 grown at 25 °C and 37 °C.

| **GO Category** | **Accession** | **Description** | **Gene names** | **Temperature (°C)** |
| --- | --- | --- | --- | --- |
| **Pathogenesis** | A1CFL1 | Alcohol dehydrogenase patD (EC 1.1.1.1) | patD ACLA_093590 | 25 |
|  | A1CFL2 | Dehydrogenase patE (EC 1.1.-.-) | patE ACLA_093600 | 25 |
|  | A4GYZ0 | Glutathione S-transferase gliG (EC 2.5.1.18) | gliG AFUA_6G09690 | 25 |
|  | A7UX13 | Hercynylcysteine sulfoxide lyase (EC 4.4.1.-) ( | egt-2 NCU11365 | 25 |
|  | B2WKF1 | Carboxypeptidase Y homolog A (EC 3.4.16.5) | cpyA PTRG_10461 | 25/37 |
|  | B6HJU2 | Glandicoline B O-methyltransferase roqN (EC 3.1.1.-) | roqN gmt Pc21g15440 | 25 |
|  | B8N406 | Secondary metabolism regulator laeA (EC 2.1.1.-) | laeA AFLA_033290 | 25 |
|  | B8NM69 | Peptidase S41 family protein ustP (EC 3.4.-.-) | ustP AFLA_095010 | 25 |
|  | B9WYE6 | Versiconal hemiacetal acetate reductase (EC 1.1.1.353) | vrdA | 25 |
|  | B9WZX1 | Tryprostatin B 6-hydroxylase (EC 1.14.13.176) | ftmP450-1 ftmC | 25 |
|  | C5FBW2 | Tripeptidyl-peptidase SED2 (EC 3.4.14.10) (Sedolisin-B) | SED2 MCYG_00184 | 25 |
|  | C5P4Z8 | Subtilisin-like protease CPC735_031240 (EC 3.4.21.-) | CPC735_031240 | 25 |
|  | D4ARB1 | Probable dipeptidyl-peptidase 5 (EC 3.4.14.-) (Dipeptidyl-peptidase V) (DPP V) (DppV) | DPP5 ARB_06651 | 25 |
|  | D4B1R0 | Probable glutamate carboxypeptidase ARB_02390 (EC 3.4.17.21) | ARB_02390 | 25 |
|  | D4B5N0 | Metallocarboxypeptidase A-like protein ARB_03789 (EC 3.4.17.-) | ARB_03789 | 25 |
|  | D7PHZ0 | Aldolase vrtJ (EC 4.1.2.-) | vrtJ | 25 |
|  | E9FCP6 | Aldo-keto reductase dtxS3 (EC 1.1.1.-) | dtxS3 MAA_10045 | 25/37 |
|  | E9QUT3 | Hydroxynaphthalene reductase arp2 (EC 1.1.-.-) | arp2 AFUA_2G17560 | 25 |
|  | J4UHQ8 | Glutathione S-transferase-like protein OpS6 (EC 2.5.1.-) | OpS6 BBA_08184 | 25 |
|  | M1W428 | Thioredoxin reductase tcpT (EC 1.8.1.-) | tcpT CPUR_02681 | 25 |
|  | M2SNN6 | Secondary metabolism regulator LAE1 (EC 2.1.1.-) | LAE1 COCHEDRAFT_1197809 | 25 |
|  | N4WHA7 | Reducing polyketide synthase PKS2 (EC 2.3.1.-) | PKS2 COCC4DRAFT_45941 | 25 |
|  | N4WQZ8 | Probable esterase TOX9 (EC 3.1.2.-) | TOX9 COCC4DRAFT_155492 | 25 |
|  | N4WW42 | Dehydrogenase RED3 (EC 1.1.1.1) | RED3 COCC4DRAFT_155403 | 25 |
|  | O42630 | Vacuolar protease A (EC 3.4.23.25) | pep2 AFUA_3G11400 | 25/37 |
|  | O43301 | Heat shock 70 kDa protein 12A | HSPA12A KIAA0417 | 25 |
|  | O74225 | Heat shock protein hsp88 | hsp88 NCU05269 | 25/37 |
|  | O74238 | Protein SnodProt1 | SNOG_13722 | 25 |
|  | O93806 | Glucosamine 6-phosphate N-acetyltransferase (EC 2.3.1.4) | GNA1 | 25 |
|  | O93866 | Heat shock 70 kDa protein | HSP70 | 25/37 |
|  | P11838 | Endothiapepsin (EC 3.4.23.22) | EAPA EPN-1 | 25 |
|  | P14010 | 4-aminobutyrate aminotransferase (EC 2.6.1.19) | gatA AN2248 | 25/37 |
|  | P15705 | Heat shock protein STI1 | STI1 YOR027W OR26.17 | 25 |
|  | P29702 | Protein farnesyltransferase/geranylgeranyltransferase type-1 subunit alpha (EC 2.5.1.58) (EC 2.5.1.59alpha) | FNTA | 25 |
|  | P29717 | Glucan 1,3-beta-glucosidase (EC 2.4.1.-) (EC 3.2.1.58) | XOG1 EXG EXG1 XOG CAALFM_C102990CA | 25 |
|  | P31540 | Heat shock protein hsp98 | hsp98 NCU00104 | 25 |
|  | P34946 | Carboxypeptidase S1 (EC 3.4.16.6) |  | 25 |
|  | P38624 | Proteasome subunit beta type-1 (EC 3.4.25.1) | PRE3 YJL001W J1407 | 25/37 |
|  | P38677 | Carboxy-cis,cis-muconate cyclase (EC 5.5.1.5) | NCU04071 | 25 |
|  | P39640 | Dihydroanticapsin 7-dehydrogenase (EC 1.1.1.385) | bacC ywfD BSU37720 ipa-82d | 25 |
|  | P40108 | Aldehyde dehydrogenase (EC 1.2.1.3) | CLAH10 CLAH3 | 25/37 |
|  | P40850 | Protein MKT1 | MKT1 YNL085W N2302 | 25/37 |
|  | P42059 | Minor allergen Cla h 7 | CLAH7 CLAH5 | 25/37 |
|  | P54006 | Protein TOXD | TOXD | 25 |
|  | P62998 | Ras-related C3 botulinum toxin substrate 1 (p21-Rac1) | RAC1 | 25 |
|  | P75863 | Uncharacterized protein YcbX | ycbX b0947 JW5126 | 25 |
|  | P78417 | Glutathione S-transferase omega-1 (GSTO-1) (EC 2.5.1.18) | GSTO1 GSTTLP28 | 25 |
|  | P79085 | Major allergen Alt a 1 | ALTA1 | 25 |
|  | P86029 | Catechol 1,2-dioxygenase (EC 1.13.11.1) | HQD2 CAALFM_C402230CA | 25 |
|  | P87017 | 5'-hydroxyaverantin dehydrogenase (EC 1.1.1.352) | aflH adhA P875_00052989-1 | 25 |
|  | P87216 | Protein vip1 | vip1 SPAC10F6.06 | 25/37 |
|  | Q00258 | Norsolorinic acid reductase A (EC 1.1.1.-) | aflE norA P875_00052990 | 25/37 |
|  | Q00278 | Norsolorinic acid ketoreductase (EC 1.1.1.349) | aflD nor-1 P875_00052988 | 25 |
|  | Q00455 | Scytalone dehydratase (EC 4.2.1.94) | SCD1 Cob_03011 | 25 |
|  | Q00859 | Mitogen-activated protein kinase (EC 2.7.11.24) | MAPK | 25/37 |
|  | Q05533 | Inositol monophosphatase 2 (EC 3.1.3.25) | INM2 IMP2 YDR287W | 25 |
|  | Q07505 | Putative carboxymethylenebutenolidase (EC 3.1.1.45) | YDL086W | 25/37 |
|  | Q0CCX5 | Questin oxidase (EC 1.-.-.-) | gedK ATEG_08459 | 25 |
|  | Q0CJ62 | 6-methylsalicylic acid decarboxylase atA (EC 1.-.-.-) | atA ATEG_06272 | 25/37 |
|  | Q12634 | Tetrahydroxynaphthalene reductase (EC 1.1.1.252) | MGG_02252 | 25 |
|  | Q2YDM1 | ADP-ribosylation factor-like protein 1 | ARL1 | 25 |
|  | Q47317 | N(6)-hydroxylysine O-acetyltransferase (EC 2.3.1.102) | iucB | 25/37 |
|  | Q49W60 | Probable nitronate monooxygenase (EC 1.13.12.16) | SSP1854 | 25 |
|  | Q4R7L8 | Peroxisomal NADH pyrophosphatase NUDT12 (EC 3.6.1.22) | NUDT12 QtsA-14876 | 25/37 |
|  | Q4V8V2 | Nucleoside diphosphate-linked moiety X motif 17 (EC 3.6.1.-) | nudt17 zgc:114128 | 25 |
|  | Q4W946 | 2-oxoglutarate-Fe(II) type oxidoreductase (EC 1.14.11.-) | encD AFUA_4G00230 | 25 |
|  | Q4WHU1 | Probable 4-hydroxyphenylpyruvate dioxygenase 1 (EC 1.13.11.27) | AFUA_2G04200 | 25/37 |
|  | Q4WMJ1 | N-methyltransferase gliN (EC 2.1.1.-) | gliN AFUA_6G09720 | 25 |
|  | Q4WMJ5 | O-methyltransferase gliM (EC 2.1.1.-) | gliM AFUA_6G09680 | 25 |
|  | Q4WMJ7 | Nonribosomal peptide synthetase gliP (EC 6.3.2.-) | gliP NRPS10 pesK AFUA_6G09660 | 25 |
|  | Q4WMJ8 | Dipeptidase gliJ (EC 3.4.13.19) | gliJ AFUA_6G09650 | 25 |
|  | Q4WMJ9 | Probable aminotransferase gliI (EC 2.6.1.-) | gliI AFUA_6G09640 | 25 |
|  | Q4WQZ7 | O-methyltransferase tpcA (EC 2.1.1.-) | tpcA tynA AFUA_4G14580 | 25/37 |
|  | Q4WZB3 | Heptaketide hydrolyase ayg1 (EC 3.7.1.-) | ayg1 AFUA_2G17550 | 25 |
|  | Q4WZS3 | Putative aspergillopepsin A-like aspartic endopeptidase AFUA_2G15950 (EC 3.4.23.-) | AFUA_2G15950 | 25 |
|  | Q54DU5 | von Willebrand factor A domain-containing protein DDB_G0292028 | DDB_G0292028 | 25 |
|  | Q5A599 | Histidine protein kinase NIK1 (EC 2.7.13.3) | NIK1 COS1 HIK1 CAALFM_C702800WA | 25 |
|  | Q5AG40 | Vacuolar protein sorting-associated protein 4 | VPS4 CAALFM_C503090WA | 25 |
|  | Q5ANB1 | White-opaque regulator 2 | WOR2 CAALFM_C305170WA | 25 |
|  | Q6F4M7 | Hydroxyquinol 1,2-dioxygenase (EC 1.13.11.37) | npcC | 25 |
|  | Q6Q875 | Oxidoreductase sirO (EC 1.1.1.-) | sirO | 25 |
|  | Q6UEF0 | NADH-dependent flavin oxidoreductase nadA (EC 1.-.-.-) | nadA P875_00053008 | 25/37 |
|  | Q6UEF1 | Oxidoreductase AflY (EC 1.-.-.-) | aflY hypA P875_00053033 | 25 |
|  | Q6WP50 | Presilphiperfolan-8-beta-ol synthase (EC 4.2.3.74) (Botrydial synthesis protein 2) | BOT2 CND15 | 25 |
|  | Q6Z965 | 12-oxophytodienoate reductase 7 (EC 1.3.1.42) | OPR7 OPR13 LOC_Os08g35740 OsJ_27573 | 25 |
|  | Q88RC0 | Glutarate-semialdehyde dehydrogenase DavD (EC 1.2.1.20) | davD PP_0213 | 25 |
|  | Q8CG76 | Aflatoxin B1 aldehyde reductase member 2 (EC 1.1.1.n11) | Akr7a2 Afar Akr7a5 | 25 |
|  | Q92250 | Farnesyl pyrophosphate synthase (EC 2.5.1.10 | fpp fpps 123A4.020 NCU01175 | 25 |
|  | Q92398 | Mitogen-activated protein kinase spm1 (EC 2.7.11.24) | spm1 pmk1 SPBC119.08 | 25/37 |
|  | Q93XW5 | Nitrile-specifier protein 5 | NSP5 At5g48180 MIF21.7 | 25 |
|  | Q96VN5 | Triosephosphate isomerase (EC 5.3.1.1) | TPI1 TPI PAAG_02585 | 25/37 |
|  | Q975C8 | Acryloyl-coenzyme A reductase (EC 1.3.1.84) | STK_04800 | 25 |
|  | Q9CA40 | Nudix hydrolase 1 (EC 3.6.1.55) | NUDT1 NUDX1 At1g68760 F14K14.13 | 25 |
|  | Q9N1F5 | Glutathione S-transferase omega-1 (EC 2.5.1.18) | GSTO1 | 25 |
|  | Q9P6C8 | Alcohol dehydrogenase 1 (EC 1.1.1.1) | adh-1 B17C10.210 NCU01754 | 25/37 |
|  | A9MYQ4 | Gamma-aminobutyraldehyde dehydrogenase (EC 1.2.1.19) | prr SPAB_01688 | 25/37 |
|  | Q9US47 | Putative succinate-semialdehyde dehydrogenase C1002.12c [NADP(+)] (EC 1.2.1.16) | SPAC1002.12c | 25 |
|  | Q9UUN9 | Aldehyde reductase 2 (EC 1.1.1.2) |  | 25 |
|  | Q9UW21 | Oxysterol-binding protein-like protein OBPalpha | OBPALPHA C5_01775C_B CaO19.10709/10710 | 25 |
|  | Q9Y885 | Putative branched-chain-amino-acid aminotransferase TOXF (EC 2.6.1.42) | TOXF | 25/37 |
|  | S0EE84 | Cytochrome P450 monooxygenase FUS8 (EC 1.-.-.-) | FUS8 FFUJ_10051 | 25 |
|  | W7MLD7 | Fusarin C synthetase (EC 2.3.1.-) | FUS1 FVEG_11086 | 25 |
|  | W7MWX4 | Putative aldehyde dehydrogenase FUS7 (EC 1.2.1.3) | FUS7 FVEG_11080 | 25 |
|  | A1CNW6 | Probable Xaa-Pro aminopeptidase ACLA_020440 (EC 3.4.11.9) | ACLA_020440 | 25/37 |
|  | B8N4P0 | Probable carboxypeptidase AFLA_037450 (EC 3.4.17.-) | AFLA_037450 | 37 |
|  | B8N8Q9 | NADPH dehydrogenase afvA (EC 1.6.99.1) | afvA AFLA_108540 | 25/37 |
|  | G3XMB9 | Ketoreductase azaE (EC 1.-.-.-) | azaE ASPNIDRAFT_212676 | 37 |
|  | G3XMC4 | Non-reducing polyketide synthase azaA (EC 2.3.1.-) | azaA ASPNIDRAFT_56946 | 37 |
|  | J4UHQ6 | Orsellinic acid synthase (EC 2.3.1.-) | OpS1 PKS9 BBA_08179 | 37 |
|  | O65679 | Probable pinoresinol-lariciresinol reductase 3 (EC 1.23.1.-) | PLR3 At4g34540 T4L20.120 | 37 |
|  | O93868 | NADP-dependent mannitol dehydrogenase (EC 1.1.1.138) | mtdH | 37 |
|  | O94246 | Putative glutamate--cysteine ligase regulatory subunit | SPCC737.06c | 25/37 |
|  | P42893 | Endothelin-converting enzyme 1 (EC 3.4.24.71) | Ece1 | 25/37 |
|  | P53619 | Coatomer subunit delta | ARCN1 COPD | 25/37 |
|  | Q01398 | Haloacetate dehalogenase H-1 (EC 3.8.1.3) | dehH1 | 37 |
|  | Q0DA50 | Zinc finger CCCH domain-containing protein 45 | Os06g0677700 LOC_Os06g46400 | 37 |
|  | Q0U6E8 | Mannitol-1-phosphate 5-dehydrogenase (EC 1.1.1.17) | mpd1 SNOG_12666 | 25/37 |
|  | Q0U6G5 | Probable Xaa-Pro aminopeptidase PEPP (EC 3.4.11.9) | PEPP SNOG_12649 | 25/37 |
|  | Q10166 | Hydrolase C26A3.11 (EC 3.5.-.-) | SPAC26A3.11 | 25/37 |
|  | Q2UpZ7 | Aspartyl aminopeptidase (EC 3.4.11.21) | dapA AO090005001447 | 37 |
|  | Q59KZ1 | Aminopeptidase 2 (EC 3.4.11.-) | APE2 CAALFM_C104400CA | 37 |
|  | Q5AK62 | Virulence protein SSD1 | SSD1 CAALFM_C504730CA | 37 |
|  | Q6ZXC1 | Probable inactive dehydrogenase easA | easA cpox3 | 25/37 |
| **Stress Response** | A1VUV0 | 2-hydroxy-6-oxo-6-phenylhexa-2,4-dienoate hydrolase (EC 3.7.1.8) | bphD Pnap_4141 | 25 |
|  | B0XPP3 | Metacaspase-1A (EC 3.4.22.-) | casA AFUB_007090 | 25 |
|  | B8N8R1 | Aromatic peroxygenase (AaP) (EC 1.11.2.1) | APO1 | 25 |
|  | B9W4V6 | Low-redox potential peroxidase (EC 1.11.1.7) | LnP | 25 |
|  | C0IW58 | Short chain dehydrogenase gsfK (EC 1.-.-.-) | gsfK | 25 |
|  | D7PI11 | O-methyltransferase gsfB (EC 2.1.1.-) | gsfB | 25 |
|  | D7PI16 | Short chain dehydrogenase gsfE (EC 1.-.-.-) | gsfE | 25 |
|  | D7PI19 | E3 ubiquitin-protein ligase TRIP12 (EC 2.3.2.26) | trip12 si:ch211-272f3.4 | 25 |
|  | F1RCR6 | LanC-like protein GCR2 | GCR2 GPCR At1g52920 F14G24.19 | 25 |
|  | F4IEM5 | Gibberellin 20-oxidase-like protein (EC 1.14.11.-) | At5g51310 MWD22.26 | 25 |
|  | F4KBY0 | Epoxide hydrolase A (EC 3.3.2.10) | ephA Rv3617 LH57_19705 | 25 |
|  | I6YGS0 | Putative monooxygenase Rv1533 (EC 1.13.12.-) | Rv1533 | 25 |
|  | O06179 | Putative alpha,alpha-trehalose-phosphate synthase [UDP-forming] 106 kDa subunit (EC 2.4.1.15) | SPAC2E11.16c SPACUNK4.16c | 25 |
|  | O14081 | Peroxiredoxin Asp f3 (EC 1.11.1.15) | aspf3 AFUA_6G02280 | 25 |
|  | O43099 | Mitogen-activated protein kinase-binding protein 1 | MAPKBP1 JNKBP1 KIAA0596 | 25 |
|  | O60336 | GTP-binding protein gtr2 | gtr2 SPCC777.05 | 25 |
|  | O74544 | Mitochondrial protein import protein mas5 | mas5 SPBC1734.11 | 25 |
|  | O74752 | GTP-binding protein rhb1 | rhb1 SPBC428.16c | 25/37 |
|  | O94363 | Serine/threonine-protein kinase srk1 (EC 2.7.11.1) | srk1 SPCC1322.08 | 25 |
|  | O94524 | Zeta-crystallin | CRYZ | 25 |
|  | O94547 | Superoxide dismutase [Mn], mitochondrial (EC 1.15.1.1) | SOD2 YHR008C | 25 |
|  | O97764 | Exportin-1 | xpo1 caf2 crm1 | 25 |
|  | P00447 | Aromatic-L-amino-acid decarboxylase (EC 4.1.1.28) | Ddc | 25/37 |
|  | P14068 | Granaticin polyketide synthase putative ketoacyl reductase 2 (EC 1.3.1.-) | gra-orf6 | 25/37 |
|  | P14173 | Allantoicase (EC 3.5.3.4) | alc-1 alc B8B8.070 NCU01816 | 25 |
|  | P16543 | Thioredoxin-1 | TRX1 TRX2 YLR043C | 25 |
|  | P18407 | Ubiquitin-activating enzyme E1 1 (EC 6.2.1.45) | UBA1 YKL210W | 25 |
|  | P22217 | Fumarate reductase 1 (FRDS1) (EC 1.3.1.6) | FRD1 FRDS FRDS1 YEL047C SYGP-ORF35 | 25 |
|  | P22515 | Peroxiredoxin PRX1, mitochondrial (EC 1.11.1.15) | PRX1 YBL064C YBL0503 YBL0524 | 25 |
|  | P32614 | Bifunctional epoxide hydrolase 2 (EC 3.3.2.10) | EPHX2 | 25 |
|  | P34227 | Fatty acid repression mutant protein 2 | FRM2 YCL026C-A YCLX08C YCLX8C | 25/37 |
|  | P34913 | Probable quinone oxidoreductase (EC 1.6.5.5) | ZTA1 YBR046C YBR0421 | 25 |
|  | P37261 | 6-phosphogluconate dehydrogenase, decarboxylating 1 (EC 1.1.1.44) | GND1 YHR183W | 25/37 |
|  | P38230 | Dihydroxy-acid dehydratase, mitochondrial (EC 4.2.1.9) | ILV3 YJR016C J1450 | 25 |
|  | P38720 | Heat shock protein 90 | hsp90 hsp1 AFUA_5G04170 | 25/37 |
|  | P39522 | scyllo-inositol 2-dehydrogenase (EC 1.1.1.370) | iolX yisS yucG yuxD BSU10850 | 25 |
|  | P40292 | Protein phosphatase 2C homolog 1 (EC 3.1.3.16) | ptc1 SPCC4F11.02 | 25/37 |
|  | P40332 | Thioredoxin | trx NCU05731 | 25 |
|  | P40371 | Cystathionine beta-synthase (EC 4.2.1.22) | cysB DDB_G0267386 | 25 |
|  | P42115 | Amine oxidase (EC 1.4.3.4) | mao | 25/37 |
|  | P46794 | 60S ribosomal protein L26-1 | RPL26A At3g49910 F3A4.4 T16K5.260 | 25 |
|  | P49253 | Thioredoxin reductase (EC 1.8.1.9) | cys-9 NCU08352 | 25/37 |
|  | P51414 | Isoflavone reductase homolog P3 (EC 1.3.1.-) | At1g75280 F22H5.17 | 25 |
|  | P51978 | Zinc finger protein ZPR1 | ZPR1 YGR211W | 25/37 |
|  | P53303 | Glutamine--fructose-6-phosphate aminotransferase [isomerizing] (GFAT) (EC 2.6.1.16) | GFA1 CAALFM_C302280CA | 25 |
|  | P53373 | 26S proteasome non-ATPase regulatory subunit 4 homolog | RPN10 MBP1 MCB1 At4g38630 F20M13.190 T9A14.7 | 25/37 |
|  | P53704 | Succinyl-CoA:3-ketoacid coenzyme A transferase 1, mitochondrial (EC 2.8.3.5) | OXCT1 OXCT SCOT | 25 |
|  | P55034 | Aldo/keto reductase slr0942 (EC 1.1.1.184) | slr0942 | 25 |
|  | P55809 | Disulfide-bond oxidoreductase YfcG (EC 1.8.4.-) | yfcG b2302 JW2299 | 25 |
|  | P74308 | Bifunctional epoxide hydrolase 2 (EC 3.3.2.10) | Ephx2 | 25 |
|  | P77526 | Epoxide hydrolase B (EC 3.3.2.10) | MT1988 | 25 |
|  | P80299 | 4-nitrophenylphosphatase (PNPPase) (EC 3.1.3.41) | pho2 SPBC15D4.15 | 25 |
|  | P95276 | O-methyltransferase MdmC (EC 2.1.1.-) | mdmC | 25/37 |
|  | Q00472 | Calcium/calmodulin-dependent protein kinase cmkA (EC 2.7.11.17) | cmkA AN2412 | 25 |
|  | Q00719 | Nitronate monooxygenase (EC 1.13.12.16) | ncd-2 G17A4.200 NCU03949 | 25/37 |
|  | Q00771 | Glutamate decarboxylase (GAD) (EC 4.1.1.15) | GAD1 YMR250W YM9920.04 | 25 |
|  | Q01284 | DNA damage tolerance protein RHC31 | AOS1 RHC31 YPR180W P9705.5 | 25/37 |
|  | Q04792 | Frataxin homolog, mitochondrial (EC 1.16.3.1) | YFH1 YDL120W | 25 |
|  | Q06624 | Beta-lactamase domain-containing protein 2 | lact-2 ZK945.1 | 25 |
|  | Q07540 | Phospholipase D1 (EC 3.1.4.4) | pld1 SPAC2F7.16c | 25 |
|  | Q09621 | Probable peptide methionine sulfoxide reductase (EC 1.8.4.11 | mxr1 SPAC29E6.05c SPAC30.09c | 25 |
|  | Q09706 | Catalase-peroxidase (EC 1.11.1.21) | katG ATEG_08422 | 25/37 |
|  | Q09859 | eIF-2-alpha kinase activator gcn1 (Translational activator gcn1) | gcn1 SPAC18G6.05c | 25 |
|  | Q0CD12 | Damage response protein 1 | DAP1 YPL170W P2515 | 25 |
|  | Q10105 | Small glutamine-rich tetratricopeptide repeat-containing protein 2 | SGT2 YOR007C UNF346 | 25/37 |
|  | Q12091 | Nucleolar protein 56 | NOP56 SIK1 YLR197W L8167.9 | 25 |
|  | Q12118 | Bleomycin hydrolase (EC 3.4.22.40) | BLMH | 25 |
|  | Q12460 | Thiopurine S-methyltransferase (EC 2.1.1.67) | tpm Nmul_A2259 | 25 |
|  | Q13867 | Oxygen-dependent coproporphyrinogen-III oxidase, mitochondrial (EC 1.3.3.3) | Cpox Cpo | 25 |
|  | Q2Y6S0 | Survival factor 1 | SVF1 FGRRES_05072 FGSG_05072 | 25 |
|  | Q3B7D0 | Cytochrome c peroxidase, mitochondrial (EC 1.11.1.5) | CCP1 FGRRES_01245 FGSG_01245 | 25 |
|  | Q4ICI6 | Mitogen-activated protein kinase HOG1 (EC 2.7.11.24) | HOG1 | 25/37 |
|  | Q4ING3 | Ribonuclease T2-like (EC 3.1.27.1) | rny1 AFUA_3G11220 | 25/37 |
|  | Q4W6D3 | 2-amino-3-carboxymuconate-6-semialdehyde decarboxylase (EC 4.1.1.45) | acmsd DDB_G0286525 | 25 |
|  | Q4WXZ5 | Heat shock protein 60 | hsp60 AN6089 | 25 |
|  | Q54LN9 | Heat shock 70 kDa protein | AN6010 | 25 |
|  | Q5B041 | Inorganic pyrophosphatase (EC 3.6.1.1) | ipp1 AN2968 | 25/37 |
|  | Q5B0C0 | Homoserine dehydrogenase (EC 1.1.1.3) | AN2882 | 25/37 |
|  | Q5B912 | Acyl-CoA ligase easD (EC 6.2.1.-) | easD AN2549 | 25/37 |
|  | Q5B998 | Quinone oxidoreductase (EC 1.6.5.5) | Cryz | 25 |
|  | Q5BA81 | Translationally-controlled tumor protein homolog | NCU06464 | 25 |
|  | Q6AYT0 | Flavohemoprotein (EC 1.14.12.17) | hmp fhp BB3091 | 25 |
|  | Q7RYV5 | Glutathione reductase (EC 1.8.1.7) | gtr-1 glr1 B10C3.130 NCU03339 | 25 |
|  | Q7WHW5 | Catalase B (EC 1.11.1.6) | catB AO090120000068 | 25/37 |
|  | Q873E8 | 2,4-dichlorophenol 6-monooxygenase (EC 1.14.13.20) | tfdB | 25/37 |
|  | Q877A8 | cAMP-dependent protein kinase regulatory subunit | pkaR AFUA_3G10000 | 25 |
|  | Q8KN28 | O-acetyl-ADP-ribose deacetylase MACROD1 (EC 3.2.2.-) (EC 3.5.1.-) | MACROD1 LRP16 | 25 |
|  | Q96UX3 | Galactinol synthase 2 (EC 2.4.1.123) | GOLS2 At1g56600 F25P12.95 | 25/37 |
|  | Q9BQ69 | Nitronate monooxygenase (EC 1.13.12.16) | PA1024 | 25 |
|  | Q9FXB2 | Glycine-rich RNA-binding protein 4, mitochondrial | RBG4 GR-RBP4 GRP4 MRBP1B At3g23830 F14O13_2 | 25 |
|  | Q9I4V0 | Uncharacterized protein C1711.08 | SPBC1711.08 | 25 |
|  | Q9LIS2 | Copper amine oxidase 1 (EC 1.4.3.21) | cao1 spao1 SPAC2E1P3.04 | 25 |
|  | Q9P782 | Glutathione S-transferase kappa 1 (EC 2.5.1.18) (GST 13-13) (GST class-kappa) (GSTK1-1) (hGSTK1) (Glutathione S-transferase subunit 13) | GSTK1 HDCMD47P | 25/37 |
|  | Q9P7F2 | Superoxide dismutase [Mn], mitochondrial (EC 1.15.1.1) | sod-2 18F11.030 NCU01213 | 25 |
|  | Q9Y2Q3 | Acyltransferase LovD (EC 2.3.1.238) | lovD | 25 |
|  | Q9Y783 | Peroxiredoxin TSA1-A (EC 1.11.1.15) | TSA1 TSA1A CAALFM_C306180CA | 25 |
|  | Q9Y7D1 | DnaJ homolog 1, mitochondrial | AN10778 | 25 |
|  | Q9Y7F0 | Thiamine thiazole synthase | sti35 FOXG_10428 | 25/37 |
|  | C8V213 | Glutathione hydrolase-like YwrD proenzyme (EC 2.3.2.2) | ywrD BSU36100 | 37 |
|  | J9N5G7 | 30 kDa heat shock protein | hsp30 NCU09364 | 37 |
|  | O05218 | Mitochondrial FAD-linked sulfhydryl oxidase ERV1 (EC 1.8.3.2) | ERV1 YGR029W | 25/37 |
|  | O74180 | Trehalose-phosphatase (EC 3.1.3.12) | TPS2 PFK3 YDR074W YD8554.07 | 37 |
|  | P19752 | Probable coatomer subunit gamma | sec21 SPAC57A7.10c | 37 |
|  | P27882 | Formate dehydrogenase (EC 1.17.1.9) | aciA AN6525 | 37 |
|  | P31688 | Curved DNA-binding protein (42 kDa protein) | cdb4 SPAC23H4.09 | 37 |
|  | P41751 | ATP-dependent (S)-NAD(P)H-hydrate dehydratase (EC 4.2.1.93) | SNOG_04206 | 37 |
|  | P85978 | Phosphatidylserine decarboxylase proenzyme 1, mitochondrial (EC 4.1.1.65) | PSD1 CAALFM_C100610WA | 37 |
|  | P87140 | THO complex subunit 4D | ALY4 DIP2 THO4D At5g37720 K12B20.19 | 25/37 |
|  | Q03134 | N-acyl-phosphatidylethanolamine-hydrolyzing phospholipase (EC 3.1.4.54) | Napepld Mbldc1 | 25/37 |
|  | Q09184 | Heat shock protein 78, mitochondrial | HSP78 CAALFM_C203390CA | 25/37 |
|  | Q0UBT2 | Stomatin-like protein 2, mitochondrial | STOML2 SLP2 HSPC108 | 25/37 |
|  | Q0UVK8 | ATP-dependent (S)-NAD(P)H-hydrate dehydratase (EC 4.2.1.93) | SNOG_04206 | 37 |
|  | Q5ABC5 | Phosphatidylserine decarboxylase proenzyme 1, mitochondrial (EC 4.1.1.65) | PSD1 CAALFM_C100610WA | 37 |
|  | Q6NQ72 | THO complex subunit 4D | ALY4 DIP2 THO4D At5g37720 K12B20.19 | 37 |
|  | Q6ZQW0 | Indoleamine 2,3-dioxygenase 2 (EC 1.13.11.-) | IDO2 INDOL1 | 37 |
|  | Q8BH82 | N-acyl-phosphatidylethanolamine-hydrolyzing phospholipase D (EC 3.1.4.54) | Napepld Mbldc1 | 37 |
|  | Q96UX5 | Heat shock protein 78, mitochondrial | HSP78 CAALFM_C203390CA | 37 |
|  | Q9UJZ1 | Stomatin-like protein 2, mitochondrial | STOML2 SLP2 HSPC108 | 37 |
