# Supplementary material for: A multi-omics analysis of the grapevine pathogen Lasiodiplodia theobromae reveals that temperature affects the expression of virulence- and pathogenicity-related genes
Source: Sci Rep. 2019 Sep 11;9:13144. doi: 10.1038/s41598-019-49551-w (PMC6739476; doi:10.1038/s41598-019-49551-w)
Supplement: Supplementary file 2 — Supplementary Information [file 41598_2019_49551_MOESM2_ESM.docx]

**A multi-omics analysis of the grapevine pathogen *Lasiodiplodia theobromae* reveals that temperature affects the expression of virulence- and pathogenicity-related genes**

Carina Félix^1^, Rodrigo Meneses^1,2^, Micael F. M. Gonçalves^1^, Laurentijn Tilleman^3^, Ana S. Duarte^1^, Jesus V. Jorrín-Novo^4^, Yves Van de Peer^2^, Dieter Deforce^3^, Filip Van Nieuwerburgh^3^, Ana C. Esteves^1, a^, Artur Alves^1,*^

One-DE original gels of extracellular medium (A/B) and mycelium (C/D) of LA-SOL3 strain grown at 25 °C (A/C) and 37 °C (B/D) for 4 days.

**
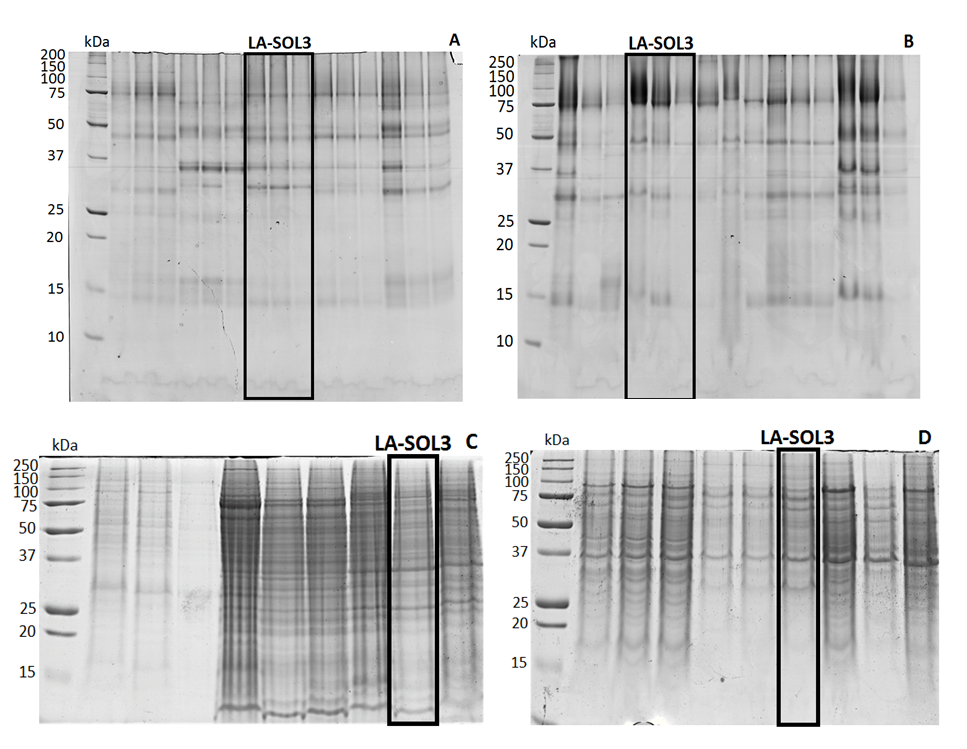
**
